# Scanning of Transposable Elements and Analyzing Expression of Transposase Genes of Sweet Potato [*Ipomoea batatas*]

**DOI:** 10.1371/journal.pone.0090895

**Published:** 2014-03-07

**Authors:** Lang Yan, Ying-Hong Gu, Xiang Tao, Xian-Jun Lai, Yi-Zheng Zhang, Xue-Mei Tan, Haiyan Wang

**Affiliations:** 1 Key Laboratory of Bio-resources and Eco-environment, Ministry of Education, Sichuan Key Laboratory of Molecular Biology and Biotechnology, Center for Functional Genomics and Bioinformatics, College of Life Sciences, Sichuan University, Chengdu, Sichuan, People’s Republic of China; 2 Chengdu Institute of Biology, Chinese Academy of Sciences, Chengdu, Sichuan, People’s Republic of China; Cankiri Karatekin University, Turkey

## Abstract

**Background:**

Transposable elements (TEs) are the most abundant genomic components in eukaryotes and affect the genome by their replications and movements to generate genetic plasticity. Sweet potato performs asexual reproduction generally and the TEs may be an important genetic factor for genome reorganization. Complete identification of TEs is essential for the study of genome evolution. However, the TEs of sweet potato are still poorly understood because of its complex hexaploid genome and difficulty in genome sequencing. The recent availability of the sweet potato transcriptome databases provides an opportunity for discovering and characterizing the expressed TEs.

**Methodology/Principal Findings:**

We first established the integrated-transcriptome database by *de novo* assembling four published sweet potato transcriptome databases from three cultivars in China. Using sequence-similarity search and analysis, a total of 1,405 TEs including 883 retrotransposons and 522 DNA transposons were predicted and categorized. Depending on mapping sets of RNA-Seq raw short reads to the predicted TEs, we compared the quantities, classifications and expression activities of TEs inter- and intra-cultivars. Moreover, the differential expressions of TEs in seven tissues of *Xushu* 18 cultivar were analyzed by using Illumina digital gene expression (DGE) tag profiling. It was found that 417 TEs were expressed in one or more tissues and 107 in all seven tissues. Furthermore, the copy number of 11 transposase genes was determined to be 1–3 copies in the genome of sweet potato by Real-time PCR-based absolute quantification.

**Conclusions/Significance:**

Our result provides a new method for TE searching on species with transcriptome sequences while lacking genome information. The searching, identification and expression analysis of TEs will provide useful TE information in sweet potato, which are valuable for the further studies of TE-mediated gene mutation and optimization in asexual reproduction. It contributes to elucidating the roles of TEs in genome evolution.

## Introduction

Sweet potato [*Ipomoea batatas*] is the world’s seventh largest food crop cultivated worldwide due to its high yield, wide adaptability and strong resistance. It is grown on about 9 million hectares in the world, yielding 140 million tons per year, and over 97% of the world output of sweet potato is produced from developing countries. The cultivated area and yield of sweet potato in China, about 6.6 million hectares and 100 million tons, account for 70% and 85% of total area and yield of the world, respectively [1], [2]. Sweet potato belongs to the *Convolvulaceae* family, *Ipomoea* genus, *Batatas* section. It is the only hexaploid (2n = 6x = 90) plant in this section with a huge genome (2,200 to 3,000 Mbp) [3]–[5] and complicated genetic structure. Many questions like genome sequencing and genetic evolution mechanism are still unresolved so far. In general, the organisms adapt to the changing environment through favorable mutation and chromosome recombination in the process of sexual reproduction. Since sweet potato mostly performs asexual reproduction, how does it reorganize its genetic substance in the process of evolution, while lacking of gametic recombination?

Transposable elements (TEs) are one of the important genetic factors for genome reorganization. They are the most abundant genomic components in eukaryotes, even accounting for more than 50% of the entire genome [6]–[9], especially in some large cereal genomes such as maize (85%), wheat (80%) and barley (84%) [10]–[13]. The TEs affect the genome as mutagenic agents by replicating and translocating to generate plasticity, producing structural changes in single gene or overall genome followed by altered spatial and temporal patterns of gene expression and, ultimately, gene function [14]. Although mutations may be harmful, and could lead to different diseases and even death of the individuals, they are the basis of biological evolution for the species. Thus, mutations generate diversity that may provide adaptive advantages to the changing environments, being further selected as a result of the natural selection [15]. The role of TEs in evolution was proposed by Barbara McClintock in the 1980s, since then progress had been made to understand the significance of TEs in genome evolution through the comprehensive study of the structure and function of TEs in different organisms [16]. For example, it has been reported that Helitrons, a kind of DNA-TEs in *Zea mays*, could capture and move gene fragments to an extent that around 20% genes in maize genome were found located differently between two maize lines [17]. And in *Arabidopsis thaliana*, Helitrons could proliferate themselves in the genome after acquiring the exon fragments [18]. Since many of the exon fragments captured by TEs are expressed, the TE-mediated exon shuffling might lead to the appearance of novel genes. A series of studies have been carried on the mutational capacity of TEs, their ability to regulate genetic systems, and their sensitivity to environmental stress. These studies have demonstrated that the TEs could not only shape the structure and function of the genomes, but also generate genetic polymorphisms favoring population adaptation, which plays a major role in genome evolution [16]. However, the TEs are still poorly understood in sweet potato as its hexaploidy genome makes genetic manipulation and genome sequencing very challenging.

In this study, we searched and identified TEs in sweet potato on the basis of the integrated-transcriptome database, which provides a large amount of expressed TE homologues. Such a large number of TEs, which were scarcely identified in sweet potato before, represent the collection of TEs with the largest number and the most complex classification in sweet potato by far. The searching, identification and expression analysis of TEs provides useful resources and information of TEs in sweet potato, which may be valuable for the study of TE-mediated gene mutation and optimization in asexual reproduction. Our result provides a new method for TE searching on species with transcriptome sequences while lacking genome information. Such amounts of TEs found in sweet potato are important data resources and material bases for studying the TE functions further. It also contributes to the elucidation of the roles of TEs in genome evolution.

## Results

### Integrated-transcriptome Database of Sweet Potato

There were totally four transcriptome databases of sweet potato which had been established from primary sequencing data of three cultivars in China. Two of them were established by our laboratory from the vegetative organ [19] and flowers [20] of *Xushu* 18 cultivar (XS 18) in 2012. The other two came from the fibrous and tuberous root of *Guangshu* 87 cultivar (GS 87) in 2010 [21] and the tuberous root of a purple sweet potato *Jingshu* 6 cultivar (JS 6) in 2012 [22], respectively. The characteristics and details of the four transcriptome databases are listed in Table 1.

**Table 1:** Characteristics and details of four primary transcriptome databases.

	XS18-v	XS18-f	GS87-r	JS6-r
Sequencing depth (folds)	49.6	Not measured	48.36	137.1
Number of reads	48,716,884	Not measured	59,233,468	25,888,890
Length of reads (bp)	75	75	75	75
Identified genes	51,763	45,698	35,051	40,280
Number of SSRs	4,249	Not measured	4,114	851
Number of Contigs	128,052	70,412	208,127	473,238
Average length of contigs (bp)	321	628	202	138
N50 (bp)	509	895	252	118

**XS18-v:** transcriptome from a mixed sample of roots, stems and leaves in cultivar *Xushu 18*. **XS18-f:** transcriptome from flowers in cultivar *Xushu 18*. **GS87-r:** transcriptome from roots in cultivar *Guangshu 87*. **JS6-r:** transcriptome from roots in cultivar *Jjingshu 6*.

Sweet potato integrated-transcriptome database was established by integrating the above four databases. All the raw reads from these databases were combined and *de novo* assembled [23]–[26]. The resulted integrated-transcriptome database comprised of totally 279,473 transcripts, 118,309 of which were >200 nt in length, and the longest one was 13,067 nt. For annotation of the assembled transcripts, sequence-similarity search was conducted against the NCBI non-redundant (Nr) protein database through Basic Local Alignment Search Tool (BLAST) alignment [27]. The transcripts longer than 200 nt were submitted for annotation through Blast2GO, and 60,976 transcripts were annotated. The integrated and the other four transcriptome databases were used for searching TEs and for analyzing the quantities, distributions and expression levels of TEs inter- and intra- cultivars, respectively.

### Prediction of TEs in the Integrated-transcriptome Database of Sweet Potato

Functional annotation and homologous sequence alignment were used to search TEs in the integrated-transcriptome database. There were totally 3,677 TEs found in 60,976 annotated transcripts using keyword searching, including 2,626 retro-TEs (class I) and 1,051 TEs (class II). Different keywords got respective results: 151 “transposon”, 659 “retrotransposon”, 432 “transposase”, 783 “reverse transcriptase”, 28 “transposable element”, 333 “retroelement”, 131 “hAT”, 92 “En/spm”, 122 “Mutator”, 71 “MULE”, 719 “Non-LTR”, 16 “PIF”, 192 “Copia”, 140 “Gypsy”, 8 “Mariner”. In addition, 1,284 TE sequences from ten kinds of other higher plants were downloaded for homologous sequence alignment with all transcripts in the integrated-transcriptome database. Among the 1284 TEs, 434 TEs got hits with similarity >70%, in which 93 got hits with similarity >90% through BLASTn with a stringent cut-off value of e-10. Among the 434 hit sequences in sweet potato integrated-transcriptome, 402 were annotated as TEs, including 106 retro-TEs and 296 TEs. As to the third searching method, 3 TEs were obtained in the pair-wise alignment between assembled sequences and the sub-terminal conserved sequence of TEs in leguminous plants with similarity >70% [28]. All above TEs were combined, the redundant TEs and the non-TE-like reverse-transcription virus were excluded. We finally identified 1,405 TEs, including 883 retro-TEs (Class I) and 522 TEs (Class II), and also found that there were 257 TEs with full length of ORF longer than 1000 nt from the Galaxy ORF prediction [29]–[30].

Among the 883 retro-TEs, there were 247 with LTR sequences which could be classified into Ty1/Copia and Ty3/Gypsy superfamily, 507 without LTR and 129 unclassified. In terms of the 522 TEs belonging to class II, 501 of them could be classified into 6 superfamilies, including Tc1/Mariner, hAT, Mutator, PIF-harbinger, CACTA and Helitron, and 21 were unclassified. The unclassified TEs showed sequence similarity lower than the threshold value set in BLAST alignment with the known TEs and thus couldn’t be classified. However, they can still be further classified if the TE-specific conserved sequences analysis was carried out. For example, the conserved sequence CTAG and its preceding 18 bp palindromic sequence was suggested to be able to produce a hairpin loop to capture gene fragments by an unknown mechanism possibly associated with their rolling circle (RC) replication process [31]. Depending on this characteristic, the initially unclassified *Arabidopsis thaliana* TE Basho was lately grouped into Helitron superfamily. This kind of TE-specific conserved sequence was also existed in the 3′ terminus of 4 unclassified sweet potato TEs. Therefore, the detailed analysis of the complete sequences and some experiment validations will be helpful to identified unclassified TEs. It is noteworthy that few of the 1,405 TEs were collected with full length in plant repeat databases like PlantTribes or Repbase [32], [33]. All the TEs identified in sweet potato are summarized in Table 2.

**Table 2:** Transposable elements identified and collected in the integrated-transcriptome database of sweet potato.

Classification		TE numbers	Length distribution (bp)	Similarity distribution
Order	Superfamily			
Retrotransposon				
LTR	Ty1/Copia	137	201–4207	51–100%
	Ty3/Gypsy	95	209–1840	46–95%
Non-LTR		507	201–6019	43–100%
Unclassified		144	201–10813	39–100%
DNA transposon				
TIR	Tc1/Mariner	6	207–926	67–77%
	hAT	136	208–3297	47–97%
	Mutator	93	204–3959	44–98%
	PIF-harbinger	23	625–2861	43–87%
	MITEs	3	416–1117	49–76%
	CACTA	80	205–3719	43–99%
Non-TIR	Helitron	159	201–3300	48–99%
IS family		4	201–1911	67–100%
Unclassified		18	201–3718	39–100%
Total		1,405	201–10813	39–100%

**Figure 1:**
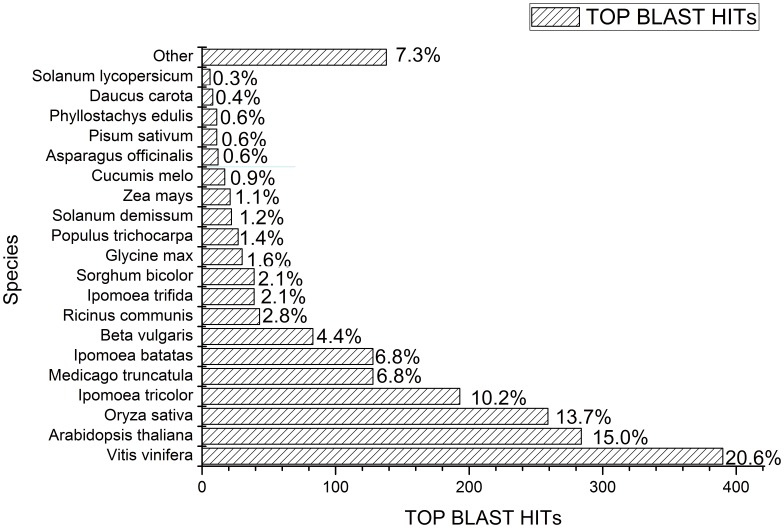
Top-Hit species distribution. 1,405 BLASTX-hit TE sequences were calculated. More than 50% of the identified TEs have the highest homology with *Vitis vinifera, Arabidopsis thaliana, Oryza sativa* and *Ipomoea tricolor*. Less than 5% of the top matches hit sweet potato itself due to the limited number of the sweet potato protein sequences available in the NCBI database.

The 1,405 TEs were submitted to GenBank for similarity alignment using BLASTn program and the result showed that the TEs in sweet potato shared deep homologies with those from at least 28 species of higher plants. The plant which got the most BLAST hits in number was *Vitis vinifera* (20.6%), followed by *Arabidopsis thaliana* (15.0%), *Oryza sativa* (13.7%), *Ipomopea trifida* (10.2%), etc (Figure 1). In addition, the plant which got the highest identity to sweet potato TEs was *Ipomopea trifida*, with sequence-similarity distribution ranging from 58% to 100%, followed by *Vitis vinifera* (50%∼98%), *Glycine max* (46%∼95%), *Populus trichocarpa* (43%∼90%), *Arabidopsis thaliana* (39%∼89%), *Zea mays* (47%∼87%), *Oryza sativa* (43%∼84%), etc. The high homologies of TEs between sweet potato and other plants revealed that these TEs were widely distributed in the vast majority of the genomes of higher plants, and thus provided further evidence for the evolutionary relationships between sweet potato and other dicotyledonous plants.

### Differences in the Number of TEs Inter- and Intra-cultivars

To compare the number of the expressed TEs in three different sweet potato cultivars and different tissues of XS 18, the reads of the four primary databases were mapped to the 1,405 TEs. Firstly, the two databases from the same XS 18 cultivar were combined, then the number of TEs among three different sweet potato cultivars (XS 18, GS 87 and JS 6) can be determined. Secondly, the two databases established from the same XS 18 cultivar, corresponding to the vegetative and reproductive organs of XS 18, were used to analyze TE expression difference and specificity between these two kinds of tissues in XS 18. Finally, the vegetative transcriptome database of XS 18 was used to analyze TE expression difference and specificity among seven different vegetative organs and tissues, including YL (young leaves), ML (mature leaves), stem, FR (fibrous roots), ITR (initial tuberous roots), ETR (expanding tuberous roots) and HTR (harvest tuberous roots).

**Figure 2:**
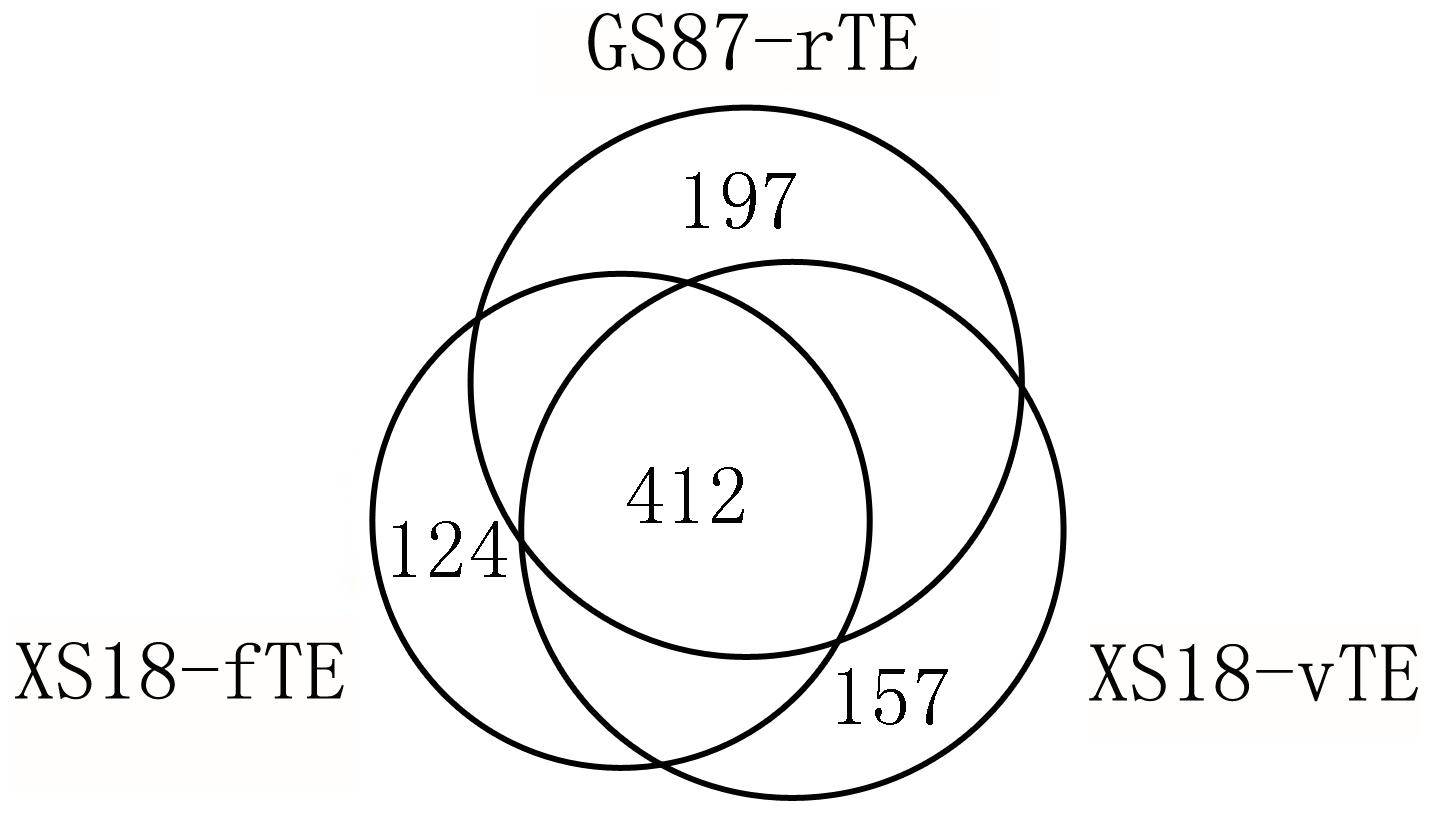
Expressed TE number in different cultivars of sweet potato. TEs were compared pair-wisely to detect the expressed TE number in different cultivars of sweet potato. Each circle represented the TE number expressed in a certain cultivar or tissue, and the cross part meant the co-expressed TE number in both two cultivars. Abbreviations GS87-rTE meant TEs in roots of cultivar *Guangshu*87, XS18-fTE meant TEs in flowers of cultivar *Xushu*18 and XS18-vTE meant TEs in vegetative organs of cultivar *Xushu*18. This overlapping circle diagram was made by auto CAD 2004 software.

**Figure 3:**
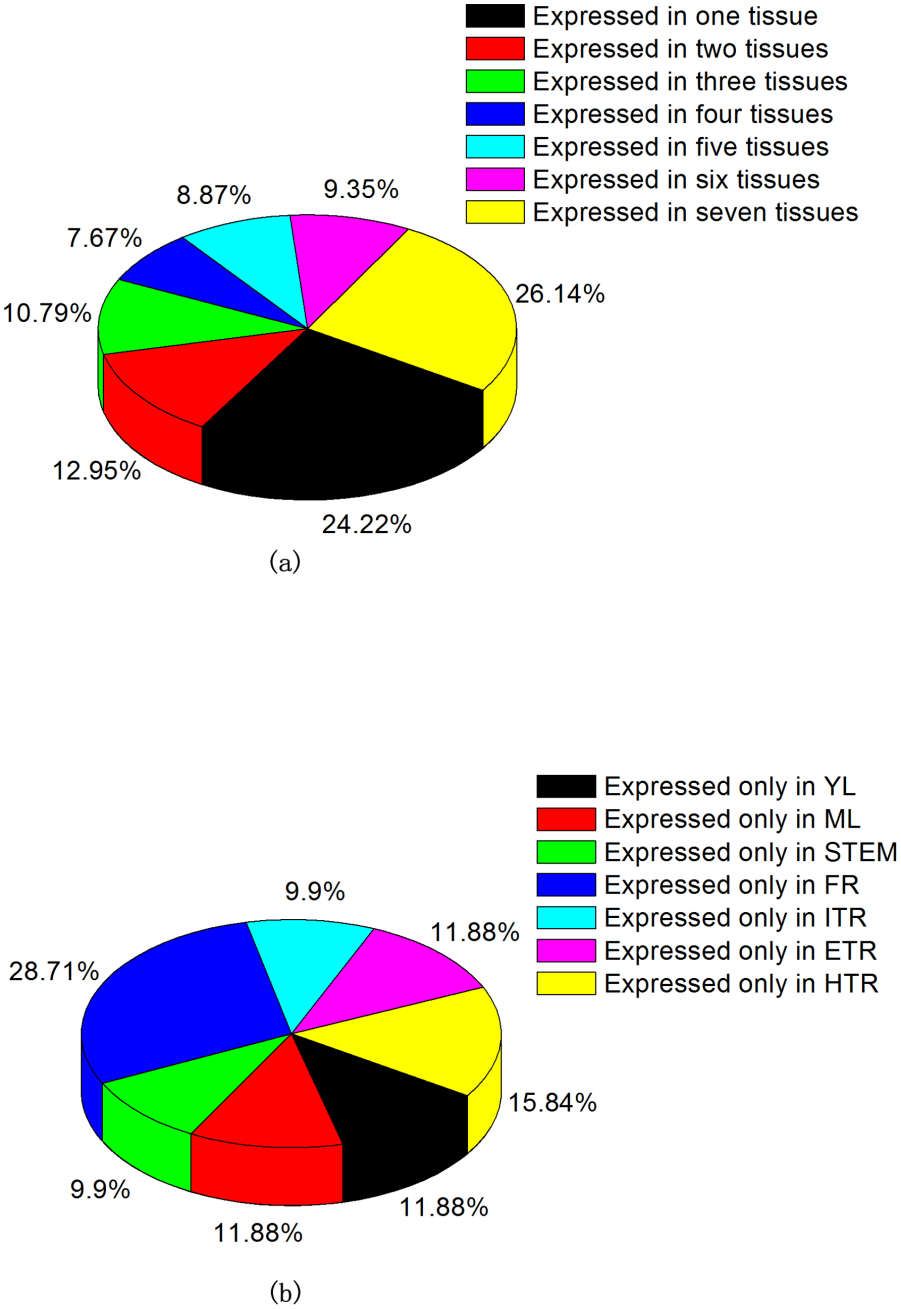
Analysis of digital gene expression (DGE) tag profiling. The analysis included (a) the statistics of the TEs expressed in 1–7 tissues. (b) the expressed TE number only in one tissues. The explanations for abbreviations were in bracket: YL (young leaves), ML (mature leaves), FR (fibrous roots), ITR (initial tuberous roots), ETR (expanding tuberous roots) and HTR (harvest tuberous roots).

Among 1,405 TEs expressed in sweet potato, there were 1,209 TEs identified in XS 18, 994 TEs in GS 87 roots and only 5 TEs in JS 6 roots. However, except for the five TEs found in JS6 which were also expressed in other two cultivars, we found that the expressions of some TEs were restricted to one cultivar. The pair-wise alignments of TEs showed that 412 TEs were specifically expressed in XS 18 (called XS18-specifically expressed TEs, XS18_SETEs) and 197 TEs in GS 87 (called GS87-specifically expressed TEs, GS87_SETEs). In the XS 18 cultivar, there were 1,030 TEs expressed in vegetative organs and 832 in reproductive organs. Similarly, there were 157 TEs expressed specifically in the vegetative organs (called vegetative organ-specifically expressed TEs, VO_SETEs) while only 124 in the reproductive organs (called reproductive organ-specifically expressed TEs, RO_SETEs) (Figure 2). The TE numbers in each of the seven vegetative organs and tissues were determined by digital gene expression (DGE) tag profiling [19]. Among 328,383 distinct clean tags generated in the DGE tag profiling, only 689 tags could be mapped to 417 TEs expressed in these seven tissues, implying that the expressions of other 988 TEs lacking the recognition bases CATG could not be detected through this method. Figure 3 demonstrated that the average number of expressed TEs in one tissue was 232. The tissue which had the lowest number of expressed TEs was YL (192 TEs), accounting for 46.04% of all the detected TEs. The highest one was FR (270 TEs), accounting for 64.75%. In addition, there were 101 TEs expressed in only one tissue, accounting for 24.22% and 109 TEs expressed in all the seven tissues, accounting for 26.14%. These two kinds of TEs were more than half of all the detected TEs and the rest TEs expressed in 2–6 tissues were relatively less (<50%). Moreover, among the tissue-specifically expressed TEs, the number expressed in FR was 2–3 times more than that in other six tissues.

### Differences in the Type of TEs Inter- and Intra-cultivars

In accordance with the above results, the types of the expressed TEs were analyzed further. The TEs expressed in each cultivar were classified into superfamilies and the results are shown in Table 3. The superfamily possessing the most number of expressed TEs in both XS 18 and GS 87 was Helitron, with 145 and 140 TEs, respectively, followed by superfamily hAT (123 and 116 TEs). Even so, there were some differences between them. In XS 18, the most abundant superfamily of retro-TEs was the Ty1/Copia, while in GS 87, Ty3/Gypsy had a slight advantage in numbers of the expressed TEs. As to the DNA-TEs, the superfamilies CACTA and PIF contained more TEs in GS 87 than in XS 18. Taken into consideration the total numbers of TEs in the two cultivars, the importance of these two superfamilies to GS87 was more obvious.

**Table 3:** Classifications of the TEs in different sweet potato cultivars.

	Retro-TEs				DNA TEs						
	Ty1/Copia	Ty3/Gypsy	Non-LTR	Unclassify	Mariner	hAT	Mutator	PIF	Helitron	CACTA	unclassify
XS18-vTE	99	68	351	53	5	115	76	19	139	60	45
XS18-fTE	76	43	319	44	3	85	59	13	88	53	50
GS87-rTE	68	76	327	48	6	116	72	20	140	70	51
JS6-rTE	0	0	2	1	0	1	1	0	0	0	0

XS18-vTE: TEs in vegetative organs of cultivar *Xushu*18. XS18-fTE: TEs in flowers of cultivar *Xushu*18. GS87-rTE: TEs in roots of cultivar *Guangshu*87. JS6-rTE: TEs in roots of cultivar *Jingshu*6.

The TE types between the vegetative and reproductive organs of XS 18 were almost the same, and the Helitron was the most abundant superfamily in both organs. The situation was the same when the TE types in seven tissues of vegetative organs in XS 18 were analyzed. The superfamily with the most expressed TEs was Helitron and the least was Ty3/Gypsy. The expressed TEs in superfamily hAT and Mutator were relatively less in the vegetative organs of XS 18 (Table 4).

**Table 4:** Statistics of the digital gene expression (DGE) tag profiling of TEs in the main superfamily.

Family name	TE members						
	YL	ML	Stem	FR	ITR	ETR	HTR
LTR-retrotransposon	17	24	17	27	14	18	21
Non-LTR retrotransposon	35	38	44	48	44	36	43
hAT transposon	28	34	32	44	37	33	39
Mutator transposon	23	29	26	25	30	27	27
PIF-harbinger transposon	7	8	7	7	6	5	9
CACTA transposon	12	18	16	16	15	12	14
Helitron transposon	37	52	54	58	35	27	59

**YL:** young leaves, **ML:** mature leaves, **FR:** fibrous roots, **ITR:** initial tuberous roots, **ETR:** expanding tuberous roots, **HTR:** harvest tuberous roots.

### Differences in the Expressing Activities of TEs Inter- and Intra-cultivars

By mapping all the reads of each cultivar transcriptome database to the identified TEs in the integrated-transcriptome database, we could calculate the relative expression level of every TE in each database and analyze the differences in the expressing activities at three levels. RPKM (reads per kilobases per million reads) was used as the standardized unit, which normalized the gene length and sequencing depth to make the expression levels of genes in different transcriptomes comparable. The comparisons among cultivars demonstrated that some TEs had stable expression levels but others showed significant differences. The major characteristics were as follow: All of the 5 TEs in JS 6 cultivar showed high expression levels, ranging from 30,000∼190,000 RPKM, much higher than their expression levels in the other two cultivars (about 2000 RPKM). As to the TEs expressed in XS 18 and GS 87, the expression levels of TEs ranged from 0.42∼70,000 RPKM and 5.59∼30,000 RPKM, respectively, indicating that the expression levels of various TEs varied greatly in different cultivars.

Meanwhile, a large number of TEs were differentially expressed in XS 18 and GS 87. For example, Ib_DTC_34571 belonging to CACTA superfamily, expressed highly in GS 87 (2759.25 RPKM) but lowly in XS 18 (0.42 RPKM). Oppositely, Ib_RN_13038, non-LTR retro-TE, had highly expression level in XS 18 (9280.42 RPKM) but lowly expressed in GS 87 (32.82 RPKM). Except for that, we also found some TEs have relatively stable expression levels in two cultivars. For example, Ib_DTM_2831 belonging to Mutator superfamily, had high and similar expression level in XS 18 (4479.14 RPKM) and GS 87 (4181.12 RPKM).

In addition, the TE expression levels between the vegetative and reproductive organs of XS 18 were compared and some TEs expressed specifically in flowers were found. For example, Ib_RU_704, unclassified retro-TE, had a high expression in flower (3243.17 RPKM) but a low expression in vegetative organs (40.09 RPKM). And Ib_RLC_116580, retro-TE belonging to LTR-Copia superfamily, was expressed highly in flower (3837.77 RPKM), but not expressed in vegetative organs. Therefore, Ib_RLC_116580 could be defined as the flower-specially expressed TE.

### DGE Analysis of TEs in Vegetative Tissues of Xushu18

**Figure 4:**
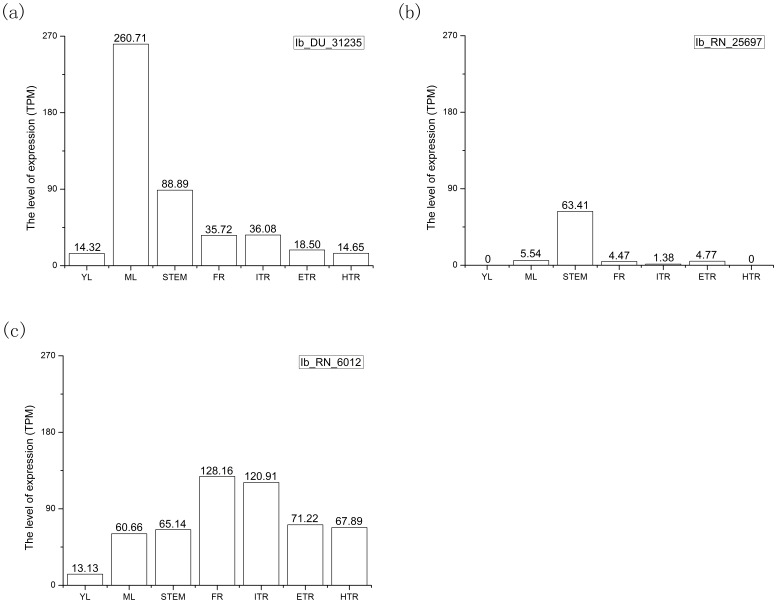
Representatives of differentially expressed TEs in different tissues and organs of *Xushu*18. (a) Differentially expressed TE Ib_DU_31235, which expressed extremely higher in ML than in other tissues. (b) Specially expressed TE Ib_RN_25697, which could not express in all tissues. (c) Differentially expressed TE Ib_RN_6012, which expressed extremely lower in YL than in other tissues. Abbreviations TPM means tags per million reads, the unit of gene expression level.

The DGE tag profiling was used to analyze TE expression levels among vegetative tissues of XS 18. We found lots of typical TEs differentially expressed in different vegetative tissues. For example, Ib_DU_31235, a unclassified TE, was expressed highly in ML (260.71 TPM, tags per million reads), but its expressions in other tissues were around 30 TPM; Ib_RN_25697, a non-LTR retro-TE, was expressed highly in stem with 63.41 TPM but lowly in other tissues (around 2 TPM), even not expressed in YL and HTR; Ib_RN_6012, a non-LTR retro-TE, was expressed lowly in YL (13.13 TPM), but highly in other 6 tissues (60.66–128.63 TPM) (Figure 4). In addition, some TEs were stably expressed in 7 tissues. For example, Ib_DTH_65331, a TE belonging to hAT superfamily, was expressed highly in seven tissues with TPM value higher than 100; Ib_DTC_11049, a TE belonging to CACTA superfamily, was expressed lowly in all tissues with TPM value around 1.

**Figure 5:**
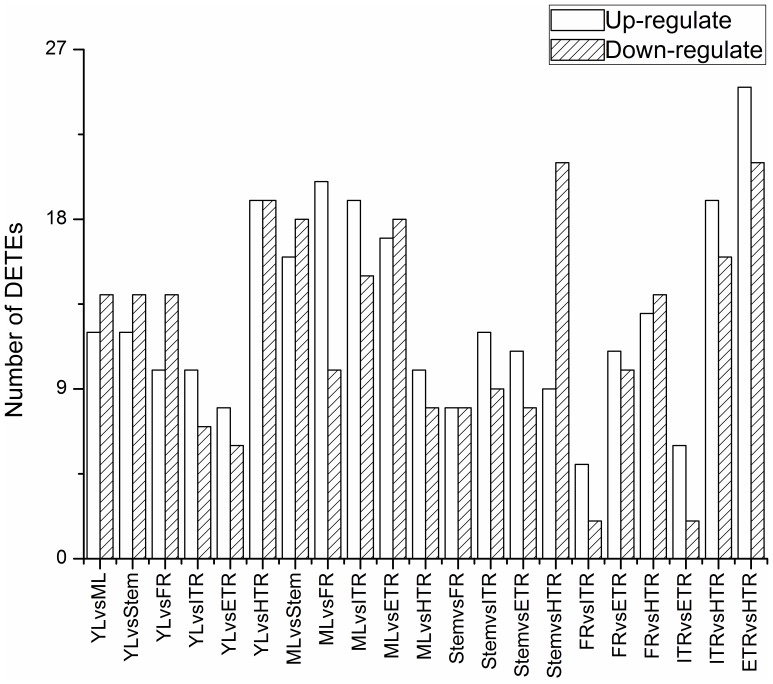
Differentially expressed TEs (DETEs) in seven tissues. Seven libraries were compared pair-wisely to detect the differentially expressed TEs between each two libraries respectively by edgeR. 21 pairs of comparison were implemented and Up-(black) and down-regulated (red) DETEs were quantified.

**Figure 6:**
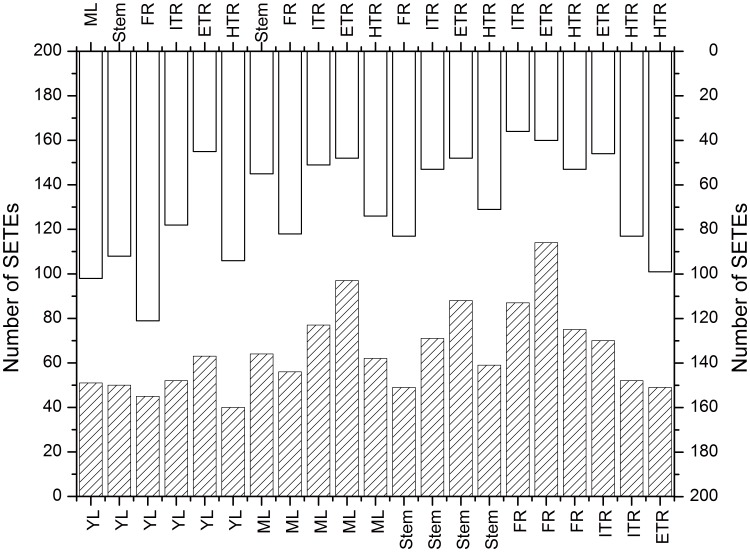
Specifically expressed TEs (SETEs) in seven tissues. Seven libraries were compared pair-wisely to detect the specifically expressed TEs between each two libraries respectively. 21 pairs of comparison were implemented and the SETEs between each two samples were quantified in red and black histograms (top vs. bottom).

To analyze differential expression patterns of TEs among seven tissues, we pair-wisely compared them and obtained 21 pairs of comparisons. There were numerous TEs showing differential expression (DETEs) and specific expression (SETEs). Among 417 TEs expressed in vegetative organs, the number of DETEs between each two tissues, including the up-regulated and the down-regulated TE, ranged from 7 to 46 and the average number was 27 (Figure 5). The largest difference was observed between ETR and HTR, and there were 25 TE transcripts up-regulated and 21 down-regulated. The smallest difference occurred between FR and ITR, in which only 7 DETEs were identified. In addition, a large number of SETEs between each two tissues were also detected. In the 21 pair-wisely comparisons, the number of SETEs which expressed in only one of the two compared tissues ranged from 108 to 166, with an average number of 137 (Figure 6). The SETE patterns among tissues revealed that the largest difference was shown between YL and FR. There were 45 TEs expressed in YL but not in FR and 121 TEs expressed in FR but not in YL, oppositely. The smallest difference occurred between YL and ETR, in which 63 and 45 TEs were specifically expressed in YL and ETR, respectively.

### Evolutionary Analysis of Transposase Genes

Sixteen TEs in sweet potato belonging to superfamily hAT, Mutator and PIF, respectively, and showing high homologies with transposase genes in other higher plants, were chosen for further studies of transposase genes. Bioinformatics analyses on these 16 TEs, involving gene length, predicted protein molecular weight, isoelectric points, etc are shown in Table S2. The coding sequences of 16 transposase genes were cloned and sequenced. The sequence similarity between the gene-clone sequences using Sanger sequencing and the assembled transcripts using Illumina sequencing were all higher than 95%, indicating that the predicted TE sequences were reliable. The comparisons between predicted and sequenced TEs are shown in Table 5.

**Table 5:** Comparison of predicted and measured transposase genes from the genome of Sweet potato.

Gene name	Predicted/measured length (bp)	Intron numbers	Intron length (bp)	BDP	ADP
Ib_DTM_FAR11812	2,475/2,475	0	\	0.28%(7/2475)	0.24%(2/824)
Ib_DTM_FAR14118	2,082/2,207	1	112	0.58%(12/2082)	0.29%(2/693)
Ib_DTM_FAR14362	2,076/2,587	2	104 and493	0.77(16/2076)	0.43%(3/692)
Ib_DTM_PB12217	2,238/2,238	0	\	0.31%(7/2238)	0.27(2/745)
Ib_DTM_PB13260	2,313/2,313	0	\	0.39%(9/2313)	0.39%(3/770)
Ib_DTM_PB14635	1,752/1,752	0	\	0.86%(15/1752)	0.86%(5/583)
Ib_DTP_9943	1,332/1,332	0	\	0.75%(10/1332)	1.12%(5/443)
Ib_DTH_1962	2,043/2,468	2	98and132	0.59%(12/2043)	0.44%(3/680)
Ib_DTM_5847	1,662/1,662	0	\	0.48%(8/1,662)	0.36% (2/553)
Ib_DTM_1664	2,538/2,754	1	209	0.55% (14/2,538)	0.59% (5/845)
Ib_DTM_2890	2,001/2,001	0	\	0.45% (9/2,001)	0.45% (3/666)
Ib_DTM_1770	2,601/2,863	1	247	0.38% (10/2,601)	0.69% (6/866)
Ib_DTM_3282	2,361/2,361	0	\	0.38% (9/2,361)	0.63% (5/786)
Ib_DTH_1235	2,712/2,889	1	168	0.59% (16/2,712)	0.66% (6/903)
Ib_DTP_11286	1,176/1,176	0	\	0.68% (8/1,176)	0.36% (3/824)
Ib_DU_2831	2,367/2,367	0	\	0.46% (11/2,367)	0.38% (3/788)

**BDP:** bases differences percentage.

**ADP:** Amino acids differences percentage.

**Figure 7:**
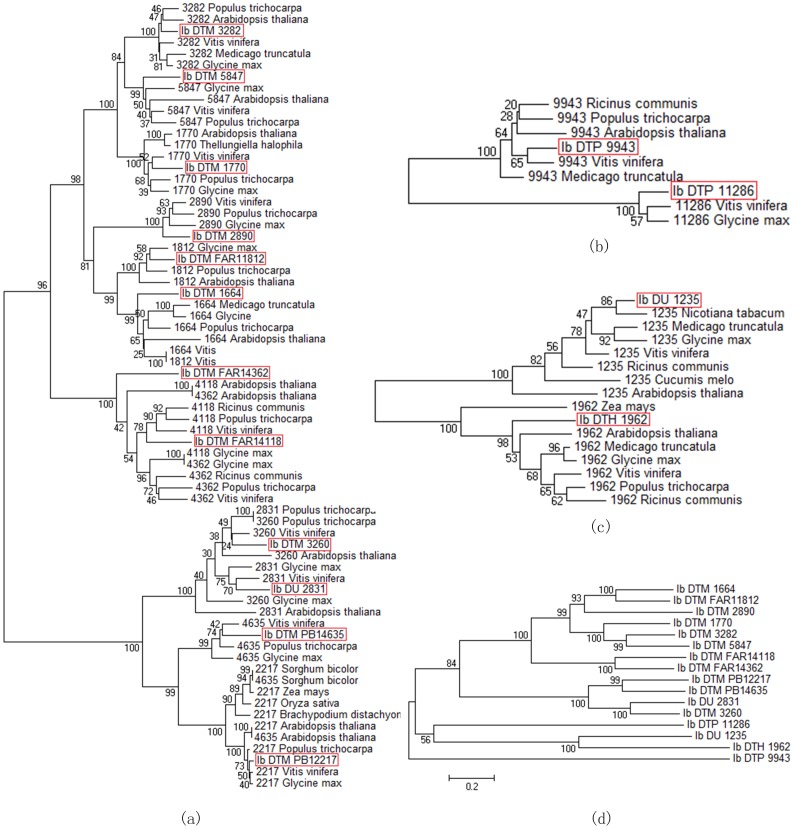
The evolutionary tree diagram of 16 transposase genes in the sweet potato. The evolutionary tree diagrams were drawn depended on the gene sequences similarity between the sweet potato and other higher plants. Each species name with an alphanumeric number means the homologous sequences in different species of each TE in sweet potato with the similarity above 80% (labeled with same digital label). The 16 sweet potato TEs were marked in red boxes. (a–c) the phylogenetic relationships of TEs in three superfamilies named Mutator, PIF-harbinger and hAT. (d) the evolutionary relationships of the 16 TEs with each other.

The 16 sequenced transposase genes were then used for evolutionary analyses. The three phylogenetic trees of TEs in three superfamilies (Figure 7, a-c) showed that there were close relationships between TEs in sweet potato and dicotyledons like *Vitis vinifera*, *Glycine max* and *Populus trichocarpa*. The TE superfamily showing the highest sequence similarity with that in *Vitis vinifera* was Mutator, such as Ib_DTM_1770, Ib_DTM_4635, etc. On the other hand, we analyzed the evolutionary relationships of 16 TEs with each other. The phylogenetic tree diagram (Figure 7d) demonstrated that the evolutionary relationships of TE in Mutator superfamily were very close, with the bootstrap of 100 between certain TEs. However, there appeared to be a clear differentiation within this family, since bootstrap 84 presented at the boundary of the two subfamilies of Mutator (Mutator-FAR and Mutator-PB1). Therefore, these Mutator-TEs could be classified in more detail. Noteworthily, Ib_DU_2831 was originally not classified, but since it was highly homologous with Ib_DTM_3260, it could be classified into Mutator superfamily. Similarly, the unclassified TE Ib_DU_1235 could be classified into hAT superfamily because of its closest genetic relationships with Ib_DTH_1962. However, from the phylogenetic tree diagram we could see that the PIF superfamily member Ib_DTP_11286 showed low homology with the same superfamily member Ib_DTP_9943, even lower than the hAT superfamily member Ib_DTH_1962. This might indicate that these two superfamilies were relatively close in sequence similarity and even in component structure. The alignment of 1,405 TEs with each other by using BLASTn was employed to analyze the genetic relationships among TEs. The results showed that 78 of 162 unclassified TEs had their relatives with similarity more than 78%, so that the evolutionary relationship analysis could be used to identify some unclassified TEs.

### Identification of Transposase-gene Copy Number

Real-time polymerase chain reaction (PCR)-based methodology for the determination of transposase-gene copy number was introduced and demonstrated [34]–[36]. Absolute quantification determines the exact copy number of transposase gene by relating the *Ct* value to a standard curve. According to the standard curves of 11 transposase genes and one single-copy S8e gene [37], the copy number of each transposase gene in genomic DNA per microliter was tested, as well as that of S8e. The transposase-gene copy number in sweet potato genome can be determined as the copy ratio of transposase gene to S8e in one sample.

**Figure 8:**
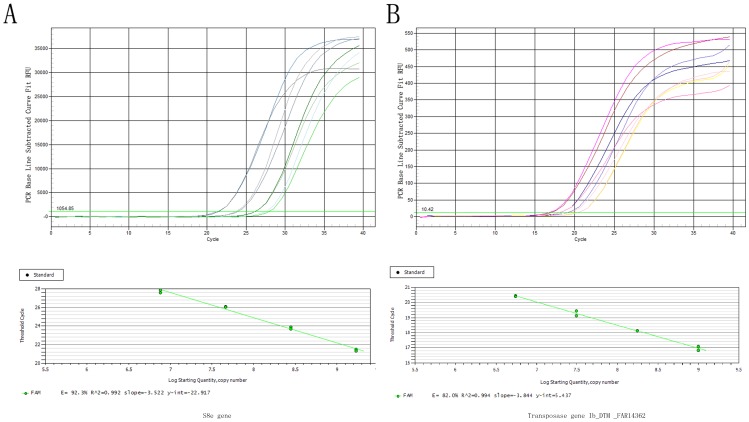
The SYBR Green I based real-time PCR for absolute quantification. Amplification graph and standard curve constructed using serially diluted DNA plasmid standards of (A) S8e gene and (B) transposase gene Ib_DTM _FAR14362, each ranging from 10^6^ to 10^9^ copies per microliter. The baseline and amplification parameters as *Ct* values in each dilution were analyzed through fluorescence data automatically.

The standard curves for Ib_DTM_FAR14362 and S8e gene, each ranging from 10^6^ to 10^9^ copies per microliter are shown in Figure 8 (the standard curves for other ten transposase genes are shown in Figure S1). The *Ct* values of Ib_DTM _FAR14362 in each dilution ranged from 16 to 21, while the ranges of *Ct* values in S8e gene were 20–28. Figure 8 shows that both curves were highly linear (R2>0.99) in the range tested by the duplicate reactions and the slopes of the standard curves were −3.84 and −3.52, respectively. From the slope of each curve, PCR amplification efficiency (E) with 0.82 and 0.92 were calculated in the investigated range, respectively. The results of absolute quantification and the calculated transposase gene copy number are shown in Table 6. It was found that the copy number was around 1 to 3. These 11 transposase genes belonged to low copy number genes, in which there were one gene with 3 copies and 6 genes with 2 copies in genome, and there were 4 genes belonging to single-copy genes. In addition, the expression levels of these 11 transposase genes in XS 18 were also calculated. They all had relatively high expression ranging from 939.33–14372.48 RPKM. As listed in Table 6, there were not any proportional relationships between gene copy number in genome and their expression levels in transcriptome.

**Table 6:** Identification of transposase gene copy number of Sweet potato using real-time PCR-based absolute quantification.

Gene name	Standards starting quantity over a dilution series(copy number)				Ct value of standards induplicate				Ct value of sample	Absolute copies/ul genomic DNA	Copies	Expression in SX18(RPKM)
S8e	7.6×10^6^	4.7×10^7^	2.8×10^8^	1.7×10^9^	27.6/27.9	26.1/26.1	23.7/23.9	21.3/21.5	26.4	2.57×10^7^	1	\
Ib_DTP_9943	5.6×10^6^	3.2×10^7^	1.8×10^8^	1.0×10^9^	20.7/21.0	19.7/19.6	18.7/18.6	17.6/17.5	19.7	2.63×10^7^	1	5,070.19
Ib_DTM_1664	5.4×10^6^	3.2×10^7^	1.7×10^8^	1.0×10^9^	20.6/19.8	19.4/19.6	18.2/18.3	16.9/18.9	18.9	5.25×10^7^	2	8,415.1
Ib_DTM _FAR11812	5.6×10^6^	3.2×10^7^	1.8×10^8^	1.0×10^9^	20.1/20.8	19.5/19.6	18.0/18.3	16.9/17.1	18.8	7.59×10^7^	3	5,439.92
Ib_DTM _FAR14362	5.4×10^6^	3.2×10^7^	1.8×10^8^	1.0×10^9^	20.4/20.5	19.1/19.5	18.1/18.1	16.8/17.1	19.0	5.25×10^7^	2	1,008.72
Ib_DTM _PB12217	5.4×10^6^	3.2×10^7^	1.8×10^8^	1.0×10^9^	23.4/23.4	22.2/22.4	21.0/21.2	19.7/20.0	22.4	2.63×10^7^	1	14,372.48
Ib_DTM _PB1 4635	5.3×10^6^	3.2×10^7^	1.7×10^8^	1.0×10^9^	23.0/22.8	22.1/21.6	20.7/20.8	19.2/19.3	21.9	2.69×10^7^	1	3,265.49
Ib_DU_2831	5.4×10^6^	3.2×10^7^	1.7×10^8^	1.0×10^9^	20.3/20.3	19.1/19.2	17.9/17.9	16.6/16.8	18.7	5.62×10^7^	2	8,816.56
Ib_DTM_2890	5.5×10^6^	3.2×10^7^	1.8×10^8^	1.0×10^9^	19.8/19.6	18.4/18.5	17.3/17.5	16.0/16.5	18.1	5.37×10^7^	2	939.33
Ib_DTM _FAR14118	5.6×10^6^	3.2×10^7^	1.8×10^8^	1.0×10^9^	20.8/20.5	19.2/19.7	17.8/17.9	16.6/16.6	18.8	5.62×10^7^	2	1,368.12
Ib_DTH_1962	5.6×10^6^	3.2×10^7^	1.8×10^8^	1.0×10^9^	20.3/19.6	18.4/18.5	17.8/17.6	16.2/16.6	18.3	5.37×10^7^	2	3,787.79
Ib_DTM_3260	5.3×10^6^	3.2×10^7^	1.7×10^8^	1.0×10^9^	21.4/21.4	20.3/20.4	19.2/19.3	17.3/17.7	20.4	2.63×10^7^	1	11,441.81

## Discussion

### A New Perspective for TEs Scanning

Transposable elements may be important motors of genetic variability, they account for the majority of genome (as in maize), and have the ability to generate genetic polymorphisms favoring population adaptation [38]. Researchers have been working to identify different TE types, in order to elucidate the molecular mechanism of TE transposition. The most widely used method to search for TE in the genome is to design element-specific primers for gene cloning by recognizing the sub-terminal conserved sequences of TEs in each family [28]. As for distinguishing different types of the TEs in model organisms, it was in accordance with their transposition chemistry, that is, by the enzymes that catalyze the DNA-strand cleavage and transfer steps necessary for their movement, such as DDE transposases in IS sequences and members of Tc/Mariner superfamily [39]. However, these methods, which detect TEs one-by-one, not only require significant labor, being costly and time-consuming, but also may miss some TE or generate false-positive clones because of genetic variations or nonspecific cloning. The availability of genome sequencing results of some model or non-model plants made genome-widely TEs prediction practical and feasible. Therefore, a few databases of plant TEs like SoyTEdb in *Glycine max* [14], as well as some integrated repetitive-element databases of eukaryotes like Repbase Update and TIGR [33], [40], were established. Therefore, the methods of TE identification in known genomes were based on the sequence alignment against the fully characterized elements in some TE databases. Along with more and more transcriptomes being sequenced, a method depending on transcriptome sequencing and gene annotation was developed, to search and identify TEs in plants without prior genome information. The advantage of this novel approach is that a global overview of potentially active TEs can be obtained. In contrast, the classic strategies required the TE to be analyzed on a one-by-one basis, being firstly identified at the DNA level, which allowed further transcription and transposition studies. For example, the 276 expressed TEs were identified in *Saccharum officinarum*, by searching from 260,781 transcriptome sequences in the sugarcane expressed-sequence tag (SUCEST) database [41]. In this paper, an improved method was used in TE searching in sweet potato, and 1,405 expressed TEs were identified after redundancy reduction. Noteworthy, the quantities and abundance of TEs searched from sweet potato were more than those searched from sugarcane. This may come from the more sensitive searching method and more abundant integrated-transcriptome database. Three different ways were used for TE searching in this study. The integrated-transcriptome database was built by re-assembling all the raw sequencing reads from four independent transcriptome databases of sweet potato representing three cultivars. It comprised plenty of transcripts with longer length and higher integrity and provided the possibilities to compare the differences of expressed TEs intra- or inter- cultivars. Some TEs were randomly selected for cloning and sequencing and the results showed that the similarities of the cloned and assembled sequences were higher than 95%, implying that the assembled TEs are considerably reliable.

Since there were few TEs in sweet potato reported before, the large numbers of TEs obtained in this study will provide an abundant resource for further studies like cloning and functional identification of interested TEs. The intensive study of their transpositions will contribute to elucidating the role of TEs in genetic variation in the process of asexual reproduction and the evolution of sweet potato.

### Prediction of the Size of TEs in the Sweet Potato Genome

Depending on transcriptome sequencing and gene annotation, 1,405 TEs were predicted from the sweet potato integrated-transcriptome through different searching methods. Even though these transcribed TE sequences could not completely reflect all the TEs existed in sweet potato genome, they represented the active part in these cultivars and tissues. Owing to the availability of the genome and transcriptome sequences of *Oryza sativa*, the TEs of rice can be predicted from the annotation information of genome and transcriptome, respectively. TEs in *Oryza sativa* genome could be acquired from the annotation information of the *Oryza sativa* genome from the web of the Rice Genome Annotation Project, http://rice.plantbiology.msu.edu/index.shtml [42]. TEs in the *Oryza sativa* transcriptome in same cultivar were obtained by employing the same method used in sweet potato (the *Oryza sativa* transcriptome were assembled ourselves and are not published). As a result, there are 17,272 TEs found in the genome and 2,021 TEs in the transcriptome,indicating that the former is about 8.6-fold more than the later and most of the TEs in the genome were silent. As to the 17,272 TEs found in rice genome, most of them could be classified into retro-TEs (12,143) and DNA-TEs (3,968). In terms of the 2,021 TEs predicted from transcriptome, there are 1,290 retro-TEs and 747 DNA-TEs. The statistics above implied that there were about 10% genomic retro-TEs and 19% genomic DNA-TEs expressed. There also existed difference of the expressed members among TE superfamilies. For example, the TE members in Ty3/Mariner superfamily identified in rice genome was twice more than that in transcriptome, meaning that less than half members were expressed. While for Non-LTR subfamily, the difference was more than one hundred times higher and the expressed TEs in this superfamily were few. Conceivably, some subfamilies, such as TNP and SNF2, were not detected to express in transcriptome. Noteworthy, for some superfamilies such as hAT and Mutator, the TE members identified in transcriptome were more than those identified in genome (85 versus 27 for hAT, and 167 versus 65 for Mutator). The TE number of these two certain superfamilies in transcriptome was 2.5–3 fold higher than that in genome, the reasons may come from the transcriptional regulation or alternative splicing, and may be associated with the discontinuity in transcriptome splicing. Even so, it is certain that the numbers and types of TEs in the genome are more than the expressed TEs in transcriptome in *Oryza sativa*. Although the ratios of TE numbers and types predicted from the genomes to those from transcriptomes showed differences among various model plant species, to a certain extent, they could be used to predict the TE numbers and types in the genome of non-model plant species on the basis of transcriptome database. At this point we can speculate that the number of TEs in sweet potato genome might be 15,000 or more.

### Diversity of Expressed TEs among Sweet Potato and other Plant Species

As for the integrated-transcriptome database of sweet potato, 3 superfamlies of retro-TEs and 7 superfamilies of DNA-TEs were identified and characterized. The distributions of TE superfamilies in transcriptomes of sweet potato and other species were compared, and some similarities among them were found. For example, Gypsy-TEs, a superfamily of LTR-retro-TEs, account for minority of retro-TEs in *Oryza sativa* (285 Gypsy-TEs in 1283 retro-TEs, about 22.2%) and the sugarcane expressed sequence tag (SUCEST) database (19 gypsy-TEs in 128 retro-TEs, about 14.8%) [41]. This is consistent with what we found in sweet potato (95 expressed Gypsy-TEs in 883 retro-TEs,10.8%). It was reported that these long terminal repeat (LTR) retro-TEs have the ability to trigger TE expression by their cis-regulatory elements in 5′ LTR. These regulatory sequences are similar to the well-characterized motifs required for the activation of stress-responsive gene expression [43]. Therefore, these TE activations caused by environmental changes could eventually result in mutagenesis in the genome, which may help the organism adapt to new environmental conditions. These TEs also played a key role in translating changes in the external environment into changes at the genomic level. Indeed, Gypsy-TEs were found to respond directly to some specific stress situations [44], but the members of this superfamily in sweet potato and other plant species was few. This is probably due to the fine transcriptional control which makes difficulty for the expression of Gypsy-TEs under normal conditions [45].

On the other hand, the expressed DNA-TEs belonging to superfamily hAT (115 TEs) and Mutator (113 TEs) accounted for 43.7% of all the DNA-TEs in sweet potato, which were similar with the distribution in other higher plants. For example, in *Arabidopsis thaliana*, the most abundant DNA-TEs in its genome were Mutator-like element (MULE), which reached 108 TEs, accounting for 17.33% among total 623 DNA-TEs [46]. It suggested that these TEs may play an important role in genome restructure [47], [48]. A process called transduplication is a potentially rich source of novel coding sequences, reflecting that the activities of these TEs have a substantial impact on the evolution of new genes in plants, by their capacity to capture and mobilize genes or fragments [49]. Just as three thousand Pack-MULEs in rice, they have mobilized fragments of more than 1,000 genes. Many of these gene fragments are likely to be non-functional pseudogenes. However, 42% of these retro-genes have recruited new exons to become chimeric genes and show some degree of function through expression [45].

However, the Non-LTR elements in retro-TEs were generally abundant in sweet potato, which is inconsistent with that in *Oryza sativa*. The reason why the Non-LTR elements were the most expressed retro-TEs in sweet potato will only be determined after a detailed analysis of their complete sequences.

### TE Distribution Differences and Environmental Adaptation

The major TE superfamilies reported in other plants are also present in three cultivars of sweet potato, but the family members and their expression levels differ enormously among cultivars. The TE number identified in *Jingshu*6 cultivar, a purple sweet potato, was extremely low, probably reflecting this cultivar may has distant genetic relationships with other two cultivars. The reasons for such big differences among cultivars need further analysis. Although the total TE numbers identified in *Xushu*18 and *Guangshu*87 were almost equal, the types and expression levels of TEs in the two cultivars were obviously different (Table 3). Specifically expressed TEs were existed in both cultivars, and differentially expressed TEs showed varied expression levels between these cultivars. To some TEs expressed in two cultivars, their expression level varied, even to the extent of hundred folds. In addition, the differentially and specifically expressed TEs were also found between the vegetative organs and reproductive organs of XS 18. There were almost 500 TEs expressed specifically within the vegetative organs while ∼200 TEs in the reproductive organs. This suggested that the expression of a TE could be changed due to the cultivars, tissues and organs.

The reason for the differential TEs expression level among cultivars may come from the environmental influences on cultivars, such as temperature and rainfall [43]. JS 6 is grown up in north china with dry climate and low rainfall, while XS 18 and GS 87 are cultivated in the south of Yangtze River with humid and rainy climate. We can speculate that the growth environment conditions of sweet potato cause the difference in the number of expressed TEs and their expression level. So the TE-induced adaptive mutations suggested a widespread role of TEs in environmental adaptation. As reported, TEs played an important role in the responsive capacity of their hosts in the face of environmental challenges [44]. For example, TEs might directly regulate the function of individual genes, provide a mechanism for rapidly acquiring new genetic material and disseminate regulatory elements that can lead to the creation of stress-inducible regulatory networks [50]. And stress-activated TEs might generate the raw diversity that species require to survive among different stressful environments. Rather than being redundant, the presence of many TEs and different expression pattern among cultivars are required to overcome the challenges imposed by different environmental conditions [43].

### Highly Expressed TE Superfamily and Genome Evolution

The result from TEs identification revealed a surprising amount of expressed TE homologues. It provided an enormous source of variability that can be used to create novel genes or modify genetic functions for genome evolution. The DGE profiling analyses demonstrated that the hAT superfamily had obvious advantages in TE expression levels, because in some highly expressed superfamilies, the expression level of TEs in Mutator was 3.40 TPM in average (the maximum value was 17.27 and the minimum value was 0.07 with 38 members), in CACTA was 1.79 TPM (the maximum was 17.16 and the minimum was 0.08 with 27 members), but in hAT it reached 42.2 TPM (the maximum was 211 and the minimum was 0.32 with 56 members). Particularly, a member (Ib_DTH_29406) of hAT superfamily had the highest average expression level in all 7 tissues of *Xushu*18. The high transcription activities of TEs in hAT superfamily in sweet potato may be related to their functional specificity. It was reported before that the hAT TEs were a diverse and ancient transposon superfamily which had insertion specificities, suggesting that they may be the most frequent contributors to genome evolution [51]. From the earliest discovered TEs like Ac in *Zea mays*, Tam in *Antirrhinum majus*, hobo in *Drosophila melanogaster* to the domesticated TEs like DAYSLEEPER in *Arabidopsis thaliana* which has been exapted for new function rather than transposition [52]–[53], these hATs has been verified to have high activity in the process of plant adaptative evolution. In addition to simply altering the gene structure, hATs insertions can lead to some positive or negative regulatory functions. For example, Ac in *Zea mays* tends to transpose into the 5′ ends of plant genes and the promoter and enhancer elements within these TEs could potentially alter gene expression [54]. No matter what the gross effects on the overall architecture of genomes caused by hAT are, or the broad range of changes in gene expression and function, from subtle quantitative effects to the rewiring of regulatory networks and the evolution of entirely new genes, it is suggesting that hAT-induced mutations has played a key part in adaptive evolution over longer periods of time in plants [55]. Therefore, whether the high expression of hAT TEs is related to the genome evolution in sweet potato should be identified in the future studies.

### High Expression Level and Low Genome Copy Number of TEs

TEs have played an important role in determining the size and structure of a complex plant genome. Every aspect of TE life cycle has the potential for genome alteration and somaclonal variation, such as increasing gene copy number and genome size, mobilization and rearrangement of gene fragments and epigenetic silencing of genes, horizontal gene transferring and chromosomal rearrangements [56]. It is crucial that TE functional characteristics are essential for explaining the dynamics and evolution of plant genomes. How the TEs acted on the various aspects of chromosome structure and evolution depended on the numbers and predominant types of TEs that expressed [57]. According to the results described above, TEs belonging to hAT and mutator superfamilies were dominant at expression level in sweet potato, but their copy number was low in the genome. The reasons for the low copy of individual transposase gene were numerous, one of them is that the total amount of TEs in Mutator and hAT is large, the copy number of each certain TE in genome should be restricted. Thus, it is not surprising that plants devote considerable resources to TE control [45].

However, the TEs in these two superfamilies generally displayed high expression and this is possibly associated with the high transposition effectiveness of the individual TE. The highly expressed TEs were predominantly active with highly mutagenic ability. And the high activity of these TEs may be useful not only for their own transposition effect, but also for providing the active components like transposase for other defective TEs with incomplete structure, such as the SINEs with huge amount and high copy number in genome. Mutation is the ultimate source of genetic variation and TEs are also likely to play a relevant role in adaptation because of their ability to generate mutations of great variety and magnitude, and their capacity to be responsive and susceptible to environmental changes [43]. From this perspective, these highly expressed but low copy number TEs could be more important than those TEs which have higher copy number and ordinary expression in the genome alteration and somatic mutation. As reported before, Mutator TEs in *Zea mays* is by far the most mutagenic plant transposon, causing new mutations at up to a hundred times the spontaneous rate [47]. The high transposition frequency and the tendency to insert into low copy sequences for such transposon have made it the primary means by which genes are mutagenized in maize (*Zea mays L.*) [48].

## Materials and Methods

### Plant Material

The sweet potato transcriptome databases used in this study were established from three cultivars in China. These cultivars were different in phenotype, planted area, main uses and so on. XS 18 is the leading cultivar in the Yangtze River Basin of China in terms of annual hectareage and total production with widely growth habit. It has green cordate and slightly toothed leaves, and elliptic roots with red skin and white flesh with purple rings in some places. It is mainly used for starch processing and the production of ethanol [58]. The second cultivar is GS 87, a local cultivar planted mostly in South China. Its vines are short, the lobed leaves are green at all stages of growth, the tuberous roots have red skin and orange flesh with good eating quality [59]. The third one is JS 6, an important food cultivar which is mainly planted in the north of China. It has spreading growth habit, green and long vines, triangular slightly lobed green leaves, and the roots are spinning and purple outside and inside [60].

Stem cuts of XS 18 were planted in May, 2011, and grown under natural light and temperature in experimental field of college of life sicences in Sichuan University, Chengdu, Sichuan Province of China. The college gave permission to conduct the study on this site and the field studies did not involve endangered or protected species. Samples from young leaves were used for PCR, RT-PCR and real-time quantitative PCR in this study. All tissue samples collected were snap-frozen immediately in nitrogen and stored at −80°C until further processing.

### Sweet Potato Transcriptome Reads Data and Databases

The reads used for assembling the sweet potato integrated transcriptome were from four transcriptome databases. The first one was the transcriptome database of vegetative organs of cultivar XS 18, including roots, stems and leaves [19]. The reads sequences were obtained in NCBI Short Read Archive (SRA, http://www.ncbi.nlm.nih.gov/Traces/sra) under accession number SRA043582, and the transcriptome sequences were in http://cfgbi.scu.edu.cn/SweetPotato/index.php. The second one was the transcriptome database of flowers of cultivar XS 18 and the reads sequences were in SRA under accession number SRA043584 [20]. The other two transcriptome databases were established from fibrous and tuberous roots of GS 87 [21] and tuberous roots of JS 6 [22], and the reads sequences were obtained all in SRA with accession number SRA022988 and SRX090758, respectively.

### Extraction of RNA and Genomic DNA

Total RNAs were extracted using the Trizol Reagent (Invitrogen, USA), and treated with DNase I (Fermentas, USA) according to the manufacturer’s instructions. RNA quality and purity were assessed with OD_230/260_ ratio. Total cDNAs were synthesized from RNA with Moloney murine leukemia virus (M-MLV) reverse transcriptase (Invitrogen, CA, USA) using oligo (dT) as primer following the manufacturer’s instructions. Genomic DNA extraction was performed using the CTAB method [61]. Agarose gel electrophoresis was used to show the integrity of the DNA, while spectrophotometry was employed to display the concentration and cleanliness.

### *De novo* Integrated Transcriptome Assembly and Annotation

All the assemblies were run on a 64-bit Linux system (Ubuntu 10.10) with 32G physical memory. Reads from four databases above were qualitatively assessed and assembled with *de novo* assemblers of Trinity (http://trinityrnaseq.sourceforge.net) [23], SOAP de novo v1.04 (http://soap.genomics.org.cn) [24] and Velvet v1.0.12 (http://www.ebi.ac.uk/~zerbino/velvet) [25] using different parameters, respectively. All of the assemblies from each assembler with optimized parameters were combined and treated with CD-HITEST to reduce redundancy (http://www.bioinformatics.org/cd-hit), and then the remains were reassembled with CAP3 (http://pbil.univ-lyon1.fr/cap3.php) [26].

### Searching for the TEs

The TEs searching was conducted in three independent screenings. Firstly, keyword searching from the annotated transcripts was used. The transcripts (>200 nt) in the sweet potato integrated-transcriptome database were submitted for annotation through BLAST using Blast2GO software v2.4.4 (http://www.blast2go.com/b2ghome) [27]. For BLASTX against the NR database, the threshold was set to E-value≤10^−5^. Based on the annotation information, TEs were retrieved from the database by keyword searching. One kind of keywords included transposon-related words, such as “transposon”, “retrotransposon”, “transposase”, “retrotransposase”, “transposable element” etc. The other kind of keywords was based on the order names and superfamily names of eukaryotic TEs, like “Non-LTR”, “Copia”, “Gypsy”, “Mutator”, “Harbinger” etc [6]. It is noteworthy that Blast2GO may be failed to give accurate annotated information when the firstly hit gene was annotated as hypothetical protein even the other hits were annotated as transposase gene. Therefore, the second method based on sequence similarity was used as a supplementary. The CDS and full-length sequences of fully characterized transposase genes of the higher plants were downloaded from GenBank (mainly from *Arabidopsis thaliana, Vitis vinifera, Ricinus communis, Glycine max, Sorghum bicolor, Oryza sativa, Populus trichocarpa, Zea mays,* etc.), and compared with transcripts in sweet potato integrated-transcriptome database using BLASTn program. Due to the enormous data and to avoid spurious matches, a very stringent expectation cut-off value (e-10 or better) was used. Thirdly, TEs were searched depending on the homology of the sub-terminal conserved sequence of TE families as reported in the literatures before [28]. All the searching results were compared pair-wisely to remove redundant, and detailed manual evaluation was further conducted to exclude the non-TEs like reverse transcription virus etc. The ORF predictions of TEs were carried out by using Galaxy (http://main.g2.bx.psu.edu/) [29], [30].

### Cloning and Sequencing of Transposase Genes

In PCR and RT-PCR reactions, primers for amplification were designed according to assembled transcripts using Primer Premier 5.0 (PREMIER Biosoft International, CA, USA) and synthesized by GENEWIZ, Inc. (http://www.genewiz.com.cn). Primer sequences are shown in Table S1. The transposase genes were amplified using KOD FX DNA polymerase (TOYOBO, Japan), under the cycling conditions as 94°C for 5 min, followed by 35 cycles consisting of 94°C for 45 s, 60°C for 45 s, and 72°C for 1 min, and a final extension cycle of 72°C for 15 min. The PCR products were fractionated and recovered in a 1% agarose gel, then ligated to 50ng vector pMD-18T (TIANGEN BIOTECH, Beijing, China) using T4 DNA ligase (TAKARA BIO, Japan). Recombinant plasmids were transformed into *Escherichia coli* JM109 competent cells [62] and clones were picked for validation through colony PCR, plasmid electrophoresis and restriction enzyme digestion (Fermentas, USA). The positive plasmids were sequenced at BGI-Shenzhen, Shenzhen, China (http://www.genomics.cn).

### Evolutionary Analysis of Transposase Gene Sequence

Transposase gene sequences of sweet potato were aligned with those of other higher plants in NCBI NR database on the bases of sequence similarity. The transposase genes from various higher plants showing similarity above 80% were screened and their sequences were downloaded from NCBI (mainly from *Vitis vinifera, Populus trichocarpa, Glycine max, Zea mays, Sorghum bicolor, Arabidopsis thaliana, Ricinus communis, Oryza sativa,* etc.). Using software CluxtalX (2.0) and MEGA4, we determined the evolutionary distance depending on the homologous differences and drew the evolutionary diagram.

### Analysis of TEs Expression in Inter- and Intra-cultivars

Expression analyses of TEs among sweet potato cultivars were carried out using the util/alignReads.pl script in Trinity software. In order to obtain the expression level of TEs in different cultivars, we aligned the reads in each of four databases to the Trinity-assembled transcripts of the integrated database. As for the expression analyses of TEs in *Xushu*18 vegetative organ, we used the DGE tag profiling [19]. According to all the tags generated from seven sequenced DGE libraries of sweet potato, including YL (young leaves), ML (mature leaves), stem, FR (fibrous roots), ITR (initial tuberous roots), ETR (expanding tuberous roots) and HTR (harvest tuberous roots), we searched CATG with the downstream 17 base pairs in the assembled TEs and the resulted 21 base pairs tags became the new expression tags related to TEs. These tags were mapped to the distinct clean tags in DGE tag profiling of transcriptome and the resulted TEs expression tags were aligned to the TE sequences using Bowtie available at the Galaxy website to detect the expression level of TEs. The edgeR package (Empirical analysis of DGE in R) was used for differential and specifical expression analysis of TEs in DGE tag profiling [63]. We normalized tag distribution for gene expression level in each library to make an effective library size and extracted differentially expressed genes (DEGs) with p value ≦0.05 and log2 fold-change ≧1. And we compared libraries pair-wise and used hypergeometric test to identify differentially expressed TEs (DETEs) and specifically expressed TEs (SETEs).

### Identification of TE Gene Copy Number

Real-time PCR-based absolute quantification was used to identify the copy number of transposase genes in sweet potato genome. The PCR standards of transposase genes and S8e gene was amplified by conventional PCR from sweet potato genomic DNA, respectively, purified using the E.Z.N.A™ Gel Extraction Kit (OMEGA, USA). The purified fragment was cloned into the TA-cloning site of a vector pMD-18T (TIANGEN BIOTECH, Beijing, China). The positive recombinant plasmids were purified using the QIAprep Spin Miniprep Kit (Qiagen) and linearized by restriction enzyme digestion. A dilution series is prepared from the cloned plasmids to provide a measure of absolute standard abundance to generate a standard curve. Linearized plasmid was quantified using a spectrophotometer and copy number was calculated for all standards by the following formula [34]–[36]:
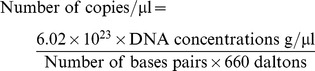



The SYBR Green based real-time PCR primer sets were designed using Beacon Designer 3.0 (Premier Biosoft International, CA). Primers stocks were prepared at 100 µM in TE (10mM Tris, pH 8.0, 1mM EDTA), and working solutions were diluted to 10 µM. All real-time PCR runs were performed in duplicate, and each reaction mixture was prepared using SYBR Premix Ex Taq kit (Takara). PCR amplifications were carried out in a total volume of 20µl, containing 6.4 µl PCR-grade water, 0.8 µl of each primer, 10 µl 2×SYBR Premix Ex Taq, and 2.0 µl appropriately diluted template DNA. It is necessary for the unknown target genes to be diluted to a point where the resulting PCR signal follows within the linear range of the standard curve, which must be determined empirically. At least 5-fold dilution may often minimizes potentially interfering substances that inhibit PCR amplification. To minimize pipetting errors and achieve better reproducibility, a master mix of the common components should be prepared and aliquoted into each well of the sample plate.

The thermal cycling protocol was as follows: initial denaturation for 10 min at 95°C followed by 40 cycles of 5 s at 95°C, 5 s at 60°C, and 5 s at 72°C. The fluorescence signal was measured at the end of each extension step at 72°C. After the amplification, a melting peak analysis with a temperature gradient of 0.1°C per second from 60 to 95°C was performed to confirm that only the specific products were amplified. Finally, the samples were cooled down to 40°C for 30s. The baseline and amplification parameters as *Ct* values in each dilution were analyzed through fluorescence data automatically. The *Ct* values were plotted against the logarithm of their initial standard copy number. Each standard curve was generated by a linear regression of the plotted points. Real-time PCR amplifications were carried on 11 transposase genes and one single copy gene S8e simultaneously from sweet potato genomic DNA with the standard dilutions in a run. Based on each standard curve, the absolute copy number of 11 unknown transposase genes from genomic DNA per µl had been derived. And then, the transposase gene copy number in sweet potato genome was calculated by dividing the copy number of transposase gene by that of S8e gene. The copy ratio of transposase gene to single-copy S8e gene equals to the transposase gene copy number. These procedures were optimized for 96-well format using a Bio-Rad IQ detection system which use fluorescein as an internal passive reference dye for normalization of well-to-well optical variation.

## Supporting Information

Figure S1 **Standard curves for 10 transposase genes.**
(PDF)

Table S1 **Sequences of primers for gene cloning.**
(XLS)

Table S2 **Bioinformatics analyses of 16 transposase genes.**
(XLS)

## References

[1] Thottappilly G (2009) Introductory Remarks. In: Loebenstein G, Thottappilly G, eds. The sweetpotato: Springer. pp 3–8.

[2] Loebenstein G (2009) Origin, Distribution and Economic Importance. In: Loebenstein G, Thottappilly G, eds. The sweetpotato: Springer. pp 9–12.

[3] Firon N, LaBonte D, Villordon A, McGregor C, Kfir Y, et al. (2009) Botany and Physiology: Storage Root Formation and Development. In: Loebenstein G, Thottappilly G, eds. The sweetpotato: Springer. pp 13–26.

[4] Gaba V, Singer S (2009) Propagation of Sweetpotatoes, In Situ Germplasm Conservation and Conservation by Tissue Culture. In: Loebenstein G, Thottappilly G, eds. The sweetpotato: Springer. pp 65–80.

[5] Srinivas T (2009) Economics of sweetpotato production and marketing. In: Loebenstein G, Thottappilly G, eds. The sweetpotato: Springer. pp 235–267.

[6] Wicker T, Sabot F, Hua-Van A, Bennetzen JL, Capy P, et al (2007) A unified classification system for eukaryotic transposable elements. Nat Rev Genet 8: 973–982. 17984973 10.1038/nrg2165

[7] International Rice Genome Sequencing Project (2005) The map-based sequence of the rice genome. Nature 436: 793–800. 16100779 10.1038/nature03895

[8] Adams MD, Celniker SE, Holt RA, Evans CA, Gocayne JD, et al (2000) The genome sequence of Drosophila melanogaster. Science 287: 2185–2195. 10731132 10.1126/science.287.5461.2185

[9] International Human Genome Sequencing Consortium (2001) Initial sequencing and analysis of the human genome. Nature 409: 860–921. 11237011 10.1038/35057062

[10] Morgante M (2006) Plant genome organisation and diversity: the year of the junk! Curr Opin Biotechnol. 17: 168–173. 10.1016/j.copbio.2006.03.001 16530402

[11] Dolgin E (2009) Maize genome mapped. Nature News 1098.

[12] The International Brachypodium Initiative (2010) Genome sequencing and analysis of the model grass Brachypodium distachyon. Nature 463: 763–768. 20148030 10.1038/nature08747

[13] The International Barley Genome Sequencing Consortium (2012) A physical, genetic and functional sequence assembly of the barley genome. Nature 491: 711–716. 23075845 10.1038/nature11543

[14] Du J, Grant D, Tian Z, Nelson RT, Zhu L, et al (2010) SoyTEdb: a comprehensive database of transposable elements in the soybean genome. BMC Genomics 11: 113. 20163715 10.1186/1471-2164-11-113 PMC2830986

[15] Yurchenkoa NN, Kovalenkoa LV, Zakharova IK (2011) Transposable Elements: Instability of Genes and Genomes. Russian Journal of Genetics: Applied Research 1: 489–496.

[16] Biemont C (2010) A brief history of the status of transposable elements: from junk DNA to major players in evolution. Genetics 186: 1085–1093. 21156958 10.1534/genetics.110.124180 PMC2998295

[17] Morgante M (2005) Massive changes of the maize genome are caused by Helitrons. Heredity 95: 421–422. 16222326 10.1038/sj.hdy.6800764

[18] Hollister JD, Gaut BS (2007) Population and evolutionary dynamics of Helitron transposable elements in Arabidopsis thaliana. Mol Biol Evol 24: 2515–2524. 17890239 10.1093/molbev/msm197

[19] Tao X, Gu YH, Wang HY, Zheng W, Li X, et al (2012) Digital gene expression analysis based on integrated de novo transcriptome assembly of sweet potato [Ipomoea batatas (L.) Lam]. PLoS One 7: e36234. 22558397 10.1371/journal.pone.0036234 PMC3338685

[20] Tao X, Gu YH, Jiang YS, Zhang YZ, Wang HY (2013) Transcriptome analysis to identify putative floral-specific genes and flowering regulatory-related genes of sweet potato. Biosci Biotechnol Biochem 77: 2169–2174. 24200775 10.1271/bbb.130218

[21] Wang Z, Fang B, Chen J, Zhang X, Luo Z, et al (2010) De novo assembly and characterization of root transcriptome using Illumina paired-end sequencing and development of cSSR markers in sweetpotato (Ipomoea batatas). BMC Genomics 11: 726. 21182800 10.1186/1471-2164-11-726 PMC3016421

[22] Xie F, Burklew CE, Yang Y, Liu M, Xiao P, et al (2012) De novo sequencing and a comprehensive analysis of purple sweet potato (Impomoea batatas L.) transcriptome. Planta 236: 101–113. 22270559 10.1007/s00425-012-1591-4

[23] Grabherr MG, Haas BJ, Yassour M, Levin JZ, Thompson DA, et al (2011) Full-length transcriptome assembly from RNA-Seq data without a reference genome. Nat Biotechnol 29: 644–652. 21572440 10.1038/nbt.1883 PMC3571712

[24] Li R, Zhu H, Ruan J, Qian W, Fang X, et al (2010) De novo assembly of human genomes with massively parallel short read sequencing. Genome Res 20: 265–272. 20019144 10.1101/gr.097261.109 PMC2813482

[25] Zerbino DR, Birney E (2008) Velvet: Algorithms for de novo short read assembly using de Bruijn graphs. Genome Res 18: 821–829. 18349386 10.1101/gr.074492.107 PMC2336801

[26] Huang X, Madan A (1999) CAP3: a DNA sequence assembly program. Genome Res 9: 868–877. 10508846 10.1101/gr.9.9.868 PMC310812

[27] Conesa A, Gotz S, Garcia-Gomez JM, Terol J, Talon M, et al (2005) Blast2GO: a universal tool for annotation, visualization and analysis in functional genomics research. Bioinformatics 21: 3674–3676. 16081474 10.1093/bioinformatics/bti610

[28] Holligan D, Zhang X, Jiang N, Pritham EJ, Wessler SR (2006) The transposable element landscape of the model legume Lotus japonicus. Genetics 174: 2215–2228. 17028332 10.1534/genetics.106.062752 PMC1698628

[29] Goecks J, Nekrutenko A, Taylor J (2010) Galaxy: a comprehensive approach for supporting accessible, reproducible, and transparent computational research in the life sciences. Genome Biol 11: R86. 20738864 10.1186/gb-2010-11-8-r86 PMC2945788

[30] Hillman-Jackson J, Clements D, Blankenberg D, Taylor J, Nekrutenko A (2012) Using Galaxy to perform large-scale interactive data analyses. Curr Protoc Bioinformatics Chapter 10: Unit10 15. 10.1002/0471250953.bi1005s38 PMC4282168 22700312

[31] Hollister JD, Gaut BS (2007) Population and evolutionary dynamics of Helitron transposable elements in Arabidopsis thaliana. Mol Biol Evol 24: 2515–2524. 17890239 10.1093/molbev/msm197

[32] Wall PK, Leebens-Mack J, Muller KF, Field D, Altman NS, et al (2008) PlantTribes: a gene and gene family resource for comparative genomics in plants. Nucleic Acids Res 36: D970–976. 18073194 10.1093/nar/gkm972 PMC2238917

[33] Jurka J, Kapitonov VV, Pavlicek A, Klonowski P, Kohany O, et al (2005) Repbase Update, a database of eukaryotic repetitive elements. Cytogenet Genome Res 110: 462–467. 16093699 10.1159/000084979

[34] Beltran J, Jaimes H, Echeverry M, Ladino Y, Lopez D, et al (2009) Quantitative analysis of transgenes in cassava plants using real-time PCR technology. In Vitro Cellular and Development Biology- Plant 45: 48–56.

[35] Yi CX, Zhang J, Chan KM, Liu XK, Hong Y (2008) Quantitative real-time PCR assay to detect transgene copy number in cotton (Gossypium hirsutum). Anal Biochem 375: 150–152. 18078801 10.1016/j.ab.2007.11.022

[36] Kihara T, Zhao CR, Kobayashi Y, Takita E, Kawazu T, et al (2006) Simple identification of transgenic Arabidopsis plants carrying a single copy of the integrated gene. Biosci Biotechnol Biochem 70: 1780–1783. 16861815 10.1271/bbb.50687

[37] Duarte JM, Wall PK, Edger PP, Landherr LL, Ma H, et al (2010) Identification of shared single copy nuclear genes in Arabidopsis, Populus, Vitis and Oryza and their phylogenetic utility across various taxonomic levels. BMC Evol Biol 10: 61. 20181251 10.1186/1471-2148-10-61 PMC2848037

[38] Schnable PS, Ware D, Fulton RS, Stein JC, Wei F, et al (2009) The B73 maize genome: complexity, diversity, and dynamics. Science 326: 1112–1115. 19965430 10.1126/science.1178534

[39] Nagy Z, Chandler M (2004) Regulation of transposition in bacteria. Res Microbiol 155: 387–398. 15207871 10.1016/j.resmic.2004.01.008

[40] Ouyang S, Buell CR (2004) The TIGR Plant Repeat Databases: a collective resource for the identification of repetitive sequences in plants. Nucleic Acids Res 32: D360–363. 14681434 10.1093/nar/gkh099 PMC308833

[41] Rossi M, Araujo PG, Van Sluys M-A (2001) Survey of transposable elements in sugarcane expressed sequence tags (ESTs). Genetics and Molecular Biology 24: 147–154.

[42] Ouyang S, Zhu W, Hamilton J, Lin H, Campbell M, et al (2007) The TIGR Rice Genome Annotation Resource: improvements and new features. Nucleic Acids Res 35: D883–887. 17145706 10.1093/nar/gkl976 PMC1751532

[43] Casacuberta E, Gonzalez J (2013) The impact of transposable elements in environmental adaptation. Mol Ecol 22: 1503–1517. 23293987 10.1111/mec.12170

[44] Bennetzen JL (2000) Transposable element contributions to plant gene and genome evolution. Plant Mol Biol 42: 251–269. 10688140

[45] Lisch D (2013) How important are transposons for plant evolution? Nat Rev Genet 14: 49–61. 23247435 10.1038/nrg3374

[46] Le QH, Wright S, Yu Z, Bureau T (2000) Transposon diversity in Arabidopsis thaliana. Proc Natl Acad Sci U S A 97: 7376–7381. 10861007 10.1073/pnas.97.13.7376 PMC16553

[47] Hershberger RJ, Benito MI, Hardeman KJ, Warren C, Chandler VL, et al (1995) Characterization of the major transcripts encoded by the regulatory MuDR transposable element of maize. Genetics 140: 1087–1098. 7672579 10.1093/genetics/140.3.1087 PMC1206663

[48] Diao XM, Lisch D (2006) Mutator transposon in maize and MULEs in the plant genome. Yi Chuan Xue Bao 33: 477–487. 16800377 10.1016/S0379-4172(06)60075-9

[49] Hoen DR, Park KC, Elrouby N, Yu Z, Mohabir N, et al (2006) Transposon-mediated expansion and diversification of a family of ULP-like genes. Mol Biol Evol 23: 1254–1268. 16581939 10.1093/molbev/msk015

[50] de Souza FS, Franchini LF, Rubinstein M (2013) Exaptation of transposable elements into novel cis-regulatory elements: is the evidence always strong? Mol Biol Evol 30: 1239–1251. 23486611 10.1093/molbev/mst045 PMC3649676

[51] Xu Z, Dooner HK (2005) Mx-rMx, a family of interacting transposons in the growing hAT superfamily of maize. Plant Cell 17: 375–388. 15659635 10.1105/tpc.104.027797 PMC548813

[52] Knip M, de Pater S, Hooykaas PJ (2012) The SLEEPER genes: a transposase-derived angiosperm-specific gene family. BMC Plant Biol 12: 192. 23067104 10.1186/1471-2229-12-192 PMC3499209

[53] Bundock P, Hooykaas P (2005) An Arabidopsis hAT-like transposase is essential for plant development. Nature 436: 282–284. 16015335 10.1038/nature03667

[54] Shimatani Z, Takagi K, Eun CH, Maekawa M, Takahara H, et al (2009) Characterization of autonomous Dart1 transposons belonging to the hAT superfamily in rice. Mol Genet Genomics 281: 329–344. 19123010 10.1007/s00438-008-0410-x PMC2758194

[55] Cowley M, Oakey RJ (2013) Transposable elements re-wire and fine-tune the transcriptome. PLoS Genet 9: e1003234. 23358118 10.1371/journal.pgen.1003234 PMC3554611

[56] Shapiro JA (2010) Mobile DNA and evolution in the 21st century. Mob DNA 1: 4. 20226073 10.1186/1759-8753-1-4 PMC2836002

[57] Tollis M, Boissinot S (2012) The evolutionary dynamics of transposable elements in eukaryote genomes. Genome Dyn 7: 68–91. 22759814 10.1159/000337126

[58] Carpena AL (2009) Important cultivars, varieties, and hybrids. In: Loebenstein G, Thottappilly G, eds. The sweetpotato: Springer. pp 27–40.

[59] Luo WL (2012) Characteristic and Measures for High Yield Cultivation of Sweet Potato Cultivar Guangshu 87 with Good Quality. Fujian Journal of Agricultural Sciences 2: 135–140.

[60] Wu QY, Qin YZ, Liu MY, Xiong XY (2011) Comparative Experiment for High-quality Sweet-potato Varieties. Hunan Agricultural Sciences 5: 4–5.

[61] He XJ, Zheng T, Su JQ (2011) ChenZD (2011) DNA Extraction of 7 Species Plant of Melastomaceae Using Modified CTAB Method. Chinese Journal of Tropical Agriculture 31: 10.

[62] Inoue H, Nojima H, Okayama H (1990) High efficiency transformation of Escherichia coli with plasmids. Gene 96: 23–28. 2265755 10.1016/0378-1119(90)90336-p

[63] Robinson MD, McCarthy DJ, Smyth GK (2010) edgeR: a Bioconductor package for differential expression analysis of digital gene expression data. Bioinformatics 26: 139–140. 19910308 10.1093/bioinformatics/btp616 PMC2796818

