# Supplementary figures and images for: Scanning of Transposable Elements and Analyzing Expression of Transposase Genes of Sweet Potato [Ipomoea batatas]
Source: PLoS One. 2014 Mar 7;9(3):e90895. doi: 10.1371/journal.pone.0090895 (PMC3946583; doi:10.1371/journal.pone.0090895)

a

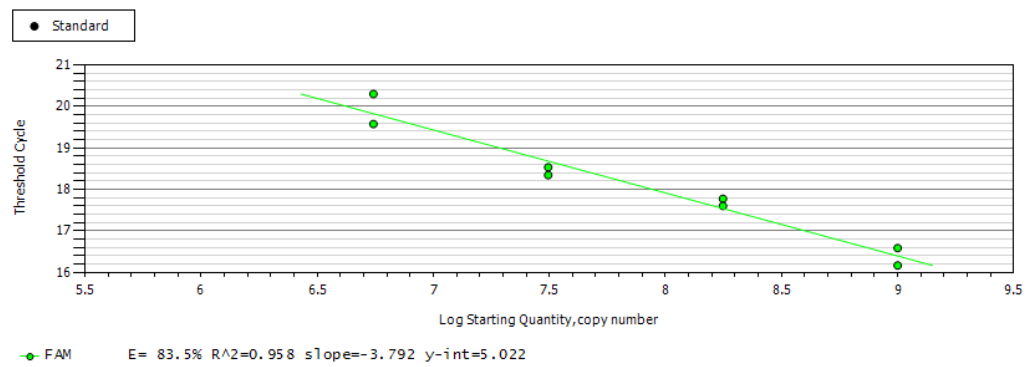

Ib\_DTH\_1962

b

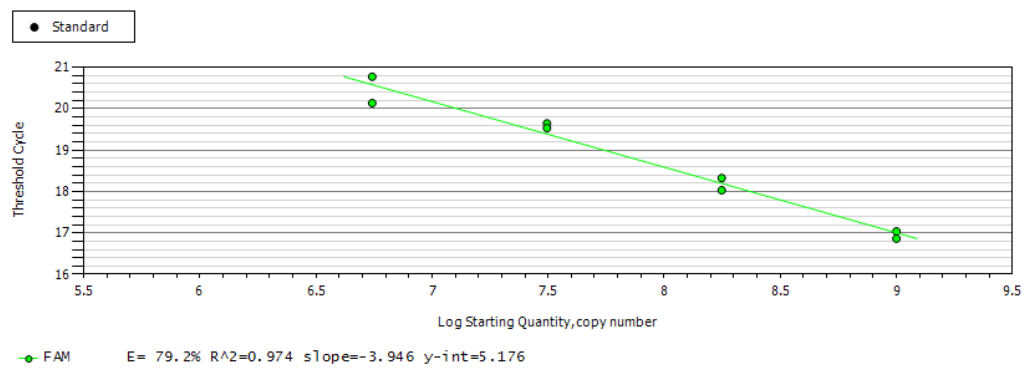

Ib\_DTM\_FAR11812

c

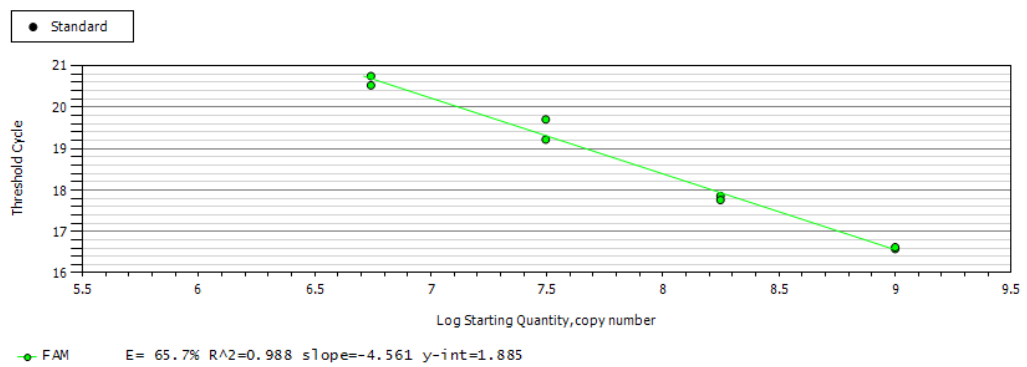

Ib\_DTM\_FAR14118

d

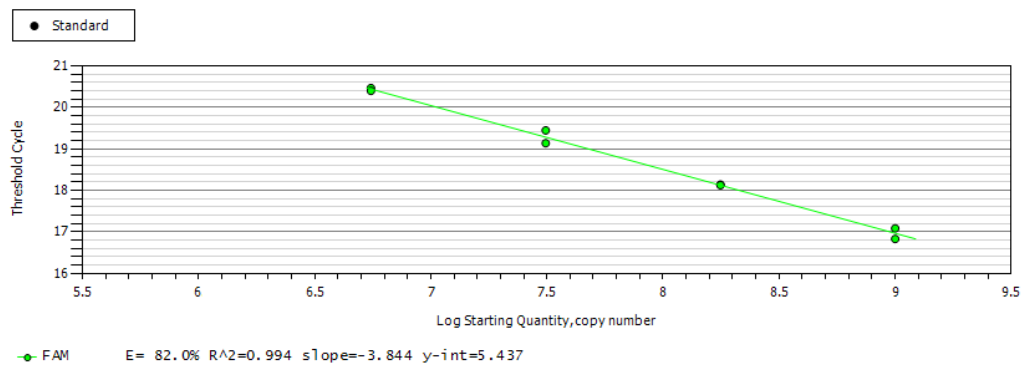

Ib\_DTM\_FAR14362

e

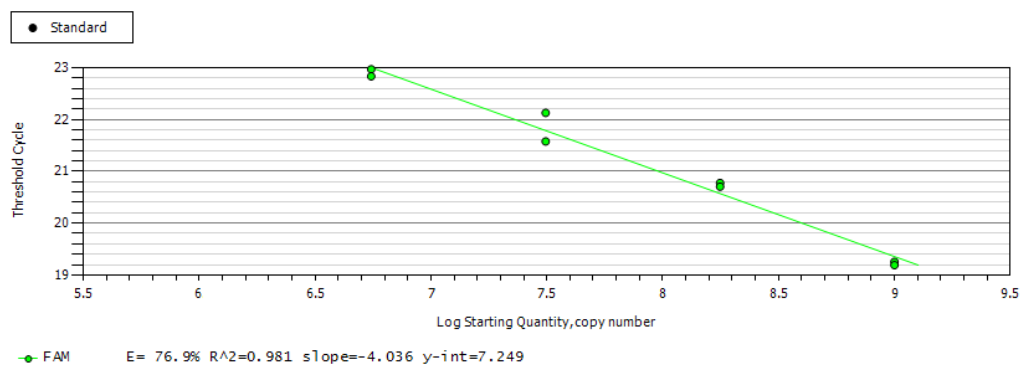

Ib\_DTM\_PB1\_4635

f

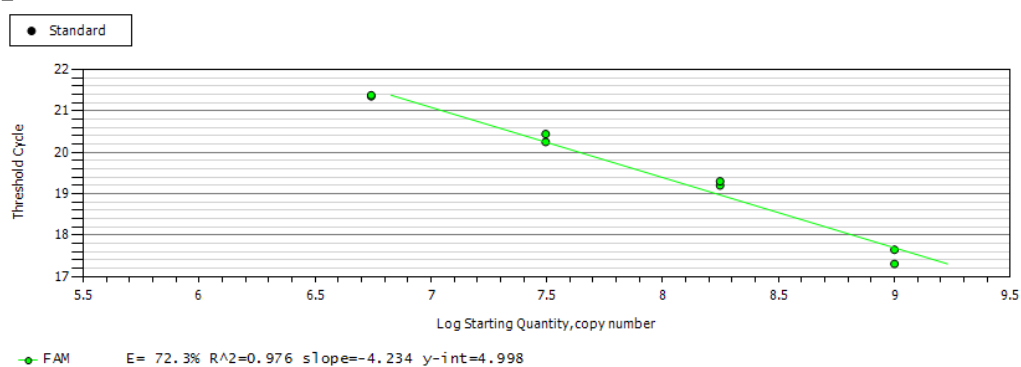

Ib\_DTM\_PB12217

g

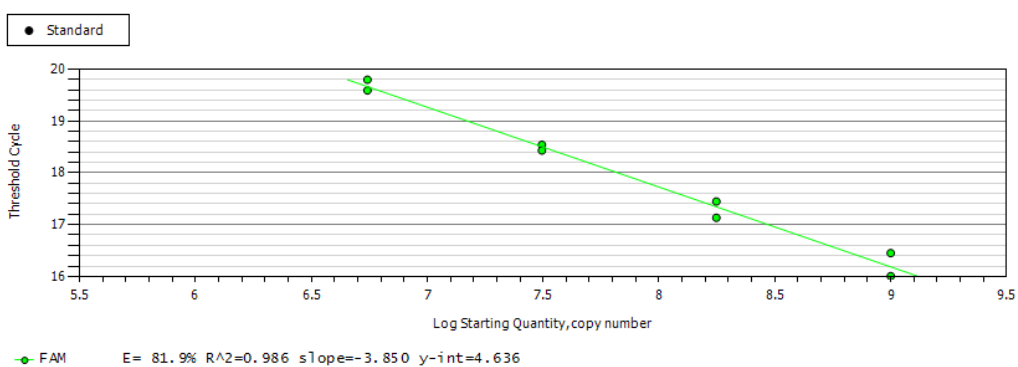

Ib\_DTM\_1664

h

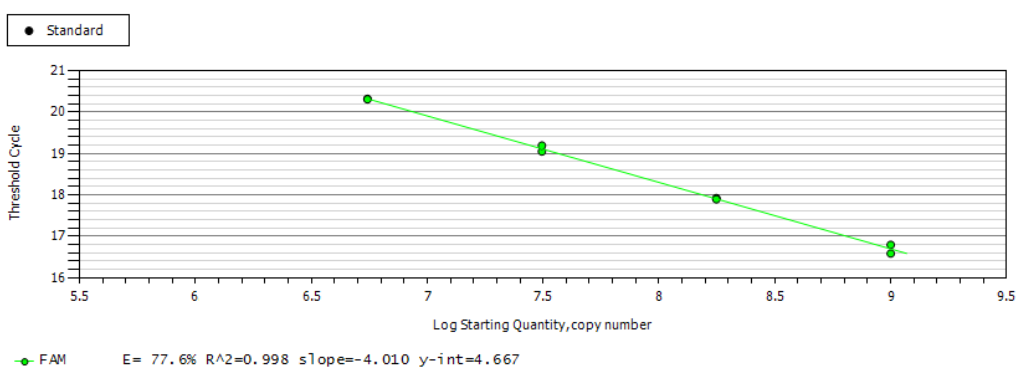

Ib\_DTM\_2890

i

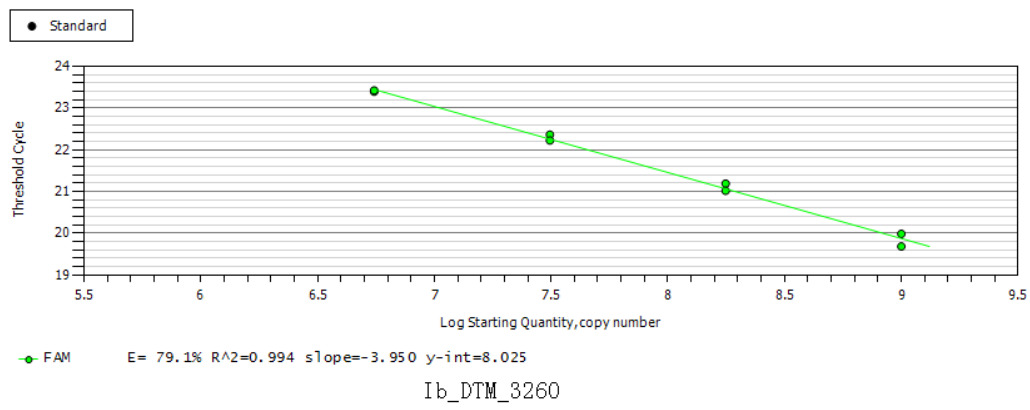

j

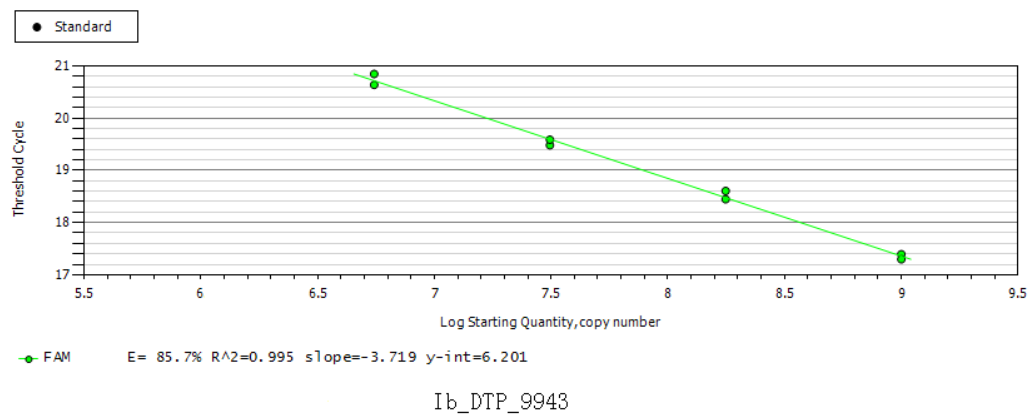

l

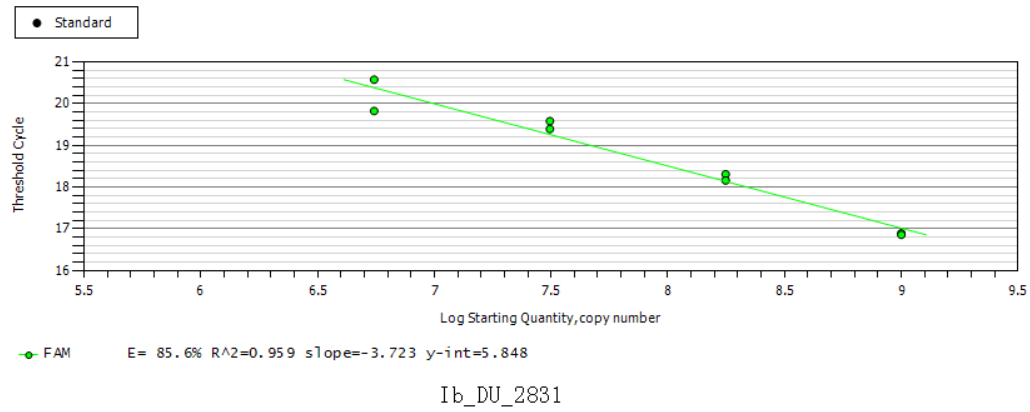

Supplement: Figure S1 — Standard curves for 10 transposase genes. (PDF) [file pone.0090895.s001.pdf]
